# *Vibrio cholerae* genomic diversity within and between patients

**DOI:** 10.1099/mgen.0.000142

**Published:** 2017-12-07

**Authors:** Inès Levade, Yves Terrat, Jean-Baptiste Leducq, Ana A. Weil, Leslie M. Mayo-Smith, Fahima Chowdhury, Ashraful I. Khan, Jacques Boncy, Josiane Buteau, Louise C. Ivers, Edward T. Ryan, Richelle C. Charles, Stephen B. Calderwood, Firdausi Qadri, Jason B. Harris, Regina C. LaRocque, B. Jesse Shapiro

**Affiliations:** ^1^​ Department of Biological Sciences, University of Montreal, Montreal, Quebec, Canada; ^2^​ Division of Infectious Diseases, Massachusetts General Hospital, Boston, MA, USA; ^3^​ Department of Medicine, Harvard Medical School, Boston, MA, USA; ^4^​ Center for Vaccine Sciences, International Centre for Diarrhoeal Disease Research, Dhaka, Bangladesh; ^5^​ National Public Health Laboratory, Ministry of Public Health and Population, Port-au-Prince, Haiti; ^6^​ Division of Global Health Equity, Brigham and Women’s Hospital, Boston, MA, USA; ^7^​ Department of Global Health and Social Medicine, Harvard Medical School, Boston, MA, USA; ^8^​ Department of Immunology and Infectious Diseases, Harvard School of Public Health, Boston, MA, USA; ^9^​ Department of Microbiology and Immunobiology, Harvard Medical School, Boston, MA, USA; ^10^​ Department of Pediatrics, Harvard Medical School, Boston, MA, USA

**Keywords:** *Vibrio cholerae*, comparative genomics, within-host evolution, horizontal gene transfer, biofilm

## Abstract

Cholera is a severe, water-borne diarrhoeal disease caused by toxin-producing strains of the bacterium *Vibrio cholerae*. Comparative genomics has revealed ‘waves’ of cholera transmission and evolution, in which clones are successively replaced over decades and centuries. However, the extent of *V. cholerae* genetic diversity within an epidemic or even within an individual patient is poorly understood. Here, we characterized *V. cholerae* genomic diversity at a micro-epidemiological level within and between individual patients from Bangladesh and Haiti. To capture within-patient diversity, we isolated multiple (8 to 20) *V. cholerae* colonies from each of eight patients, sequenced their genomes and identified point mutations and gene gain/loss events. We found limited but detectable diversity at the level of point mutations within hosts (zero to three single nucleotide variants within each patient), and comparatively higher gene content variation within hosts (at least one gain/loss event per patient, and up to 103 events in one patient). Much of the gene content variation appeared to be due to gain and loss of phage and plasmids within the *V. cholerae* population, with occasional exchanges between *V. cholerae* and other members of the gut microbiota. We also show that certain intra-host variants have phenotypic consequences. For example, the acquisition of a *Bacteroides* plasmid and non-synonymous mutations in a sensor histidine kinase gene both reduced biofilm formation, an important trait for environmental survival. Together, our results show that *V. cholerae* is measurably evolving within patients, with possible implications for disease outcomes and transmission dynamics.

## Abbreviations

HGT, horizontal gene transfer; HK, histidine kinase; hqSNV, high quality single-nucleotide polymorphism; ICE, Integrative Conjugative Element; iSNV, intra-host single nucleotide variant; MRCA, most recent common ancestor; NS, non-synonymous; S, synonymous.

## Data Summary

1. Illumina sequence data have been deposited in the NCBI Sequence Reads Archive (SRA) under BioProject PRJNA400505. Individual sample accession numbers are listed in Table S1 (available in the online version of this article).

2. Assemblies are available from a GitHub repository (https://github.com/ilevade/Vibrio_cholerae_within_patient_assemblies).

## Impact Statement

Certain bacterial pathogens can evolve and diversify within the human host, often altering virulence and antibiotic resistance. However, most examples of within-host evolution have come from chronic infections, in which the pathogen has sufficient time to mutate and diversify, and little attention has been paid to more acute infections such as the one caused by *V. cholerae*. By sequencing multiple bacterial isolates from each of eight patients from Bangladesh and Haiti, we found that cholera patients can harbour a diverse population of *V. cholerae.* As expected for an acute infection, this diversity is limited, ranging from zero to three point mutations (single nucleotide variants) per patient. However, gene gain/loss events are more prevalent than point mutations, occurring in every single patient, and sometimes involving the transfer of dozens of genes on plasmids. Even if rare, point mutations and gene gain/loss events may be maintained by natural selection, and can alter clinically- and environmentally-relevant phenotypes such as biofilm formation. Therefore, within-patient evolution has the potential to impact clinical and epidemiological outcomes. Together, our results demonstrate that within-patient evolution may be a general feature of both acute and chronic infections, and that gene gain/loss may be an important feature of within-host evolution.

## Introduction

Cholera is an acute diarrhoeal infection that remains a serious health threat in countries with limited access to clean water [1]. *Vibrio cholerae* is the causative agent of the disease and is a natural inhabitant of aquatic ecosystems [2], with more than 200 serogroups identified to date on the basis of their somatic O antigens [3, 4]. Most *V. cholerae* serogroups are not pathogenic; only isolates in serogroup O1 (consisting of two biotypes known as ‘classical’ and ‘El Tor’ and the serotypes Ogawa and Inaba) and O139 have been identified as agents of cholera epidemics and pandemics [1].

Whole genome sequencing and population genomics have the potential to improve our understanding of the epidemiology, aetiology and evolution of bacterial infectious diseases [5]. For example, comparisons of whole-genome sequences of strains of *V. cholerae* from across the world, over the course of a century, clarified the history of the current pandemic [6] and showed that this pandemic is the result of a single clonal expansion of one *V. cholerae* O1 El Tor ancestor, accompanied by horizontal gene transfer (HGT) events involving toxin and antibiotic resistance genes [7]. More recently, comparative genomics has been applied to answer epidemiological questions, proving the Asian origin of the strain causing the ongoing Haitian cholera outbreak, which began in 2010 [8–11]. Using whole genome sequencing and single nucleotide polymorphism (SNP) analysis, Azarian *et al*. [12] compared 60 clinical and environmental isolates collected in Haiti from 2010 to 2012. They found that the 2011 and 2012 strains rapidly diverged from the 2010 ancestral strain that initiated the outbreak, suggesting evolution driven by positive selection in a new environment [12].

Viral pathogens can evolve and diversify within infected patients, with serious consequences for disease outcome [13], and certain bacterial pathogens have recently been shown to diversify within patients as well [14]. However, evolutionary and epidemiological studies have conventionally been conducted with just one bacterial isolate taken as representative of the infection, even though within-patient diversity is important to consider, for several reasons [15–18]. Within-host evolution may impact the longer-term evolution and transmission potential of pathogens, particularly if there are fitness trade-offs between evolution within and between hosts. For example, a study of one cholera patient from Haiti showed that phage-resistant *V. cholerae* mutants rose to high frequency within the patient due to positive selection imposed by phage predation [19]. The study showed how strong selection can shape *V. cholerae* diversity within patients, but the prevalence and extent of *V. cholerae* genetic diversity within patients remains unclear and also whether intra-host evolution is generally driven by selection.

As for many other bacterial pathogens, the prevailing orthodoxy is that *V. cholerae* infections are essentially clonal, and essentially devoid of within-host genetic diversity. Although within-host populations of *V. cholerae* have not been studied extensively, evidence suggests that within-host diversity does indeed exist, at the level of phase variation in the O antigen, or in variable number tandem repeat (VNTR) loci [4, 20]. This diversity could arise by within-host evolution, or be due to infection by different strains that diverged before entering the host. *V. cholerae* is genetically diverse in aquatic ecosystems [21] and co-infections from diverse environmental strains are possible [22]. *V. cholerae* infections are acute, lasting only a few days before the patient either recovers or dies [1]. Therefore, there is limited time for within-host evolution (including mutation, recombination and selection) to occur. On the other hand, measurable within-host evolution has been demonstrated over just 6 days in a *Burkholderia pseudomallei* infection [23]. It is therefore expected that even if *V. cholerae* will experience less within-host evolution compared to more chronic bacterial infections with documented within-host evolution [24–29], diversity among isolates from the same patient could be detectable. Indeed, *V. cholerae* grows to large population sizes within the host (from 10^7^ to 10^9^ vibrios per gram of stool), dominating the gut microbiome [30, 31]. If the effective population size within a host is large, many mutations are expected and natural selection will be efficient. However, *V. cholerae* likely experiences population bottlenecks upon infection and within the gut [32], which would reduce genetic diversity and reduce the efficiency of selection. In addition to point mutations, *V. cholerae* can undergo high rates of HGT [7, 33, 34], providing an additional potential source of within-host diversity. During an infection, *V. cholerae* could acquire genes from plasmids, phages, pathogenicity islands or genes from the gut microbiota, which appears to be a hot-spot of HGT [35]. However, the extent of within-patient mutation, HGT and natural selection are still poorly known for *V. cholerae*.

In this study, we characterized genomic diversity of *V. cholerae* within and between eight cholera patients, sequencing between eight and 20 isolate genomes per patient. We identified both intra-host single nucleotide variants (iSNVs) and gene gain/loss events within patients. As expected for an acute infection, few within-patient point mutations were detected, ranging from zero to three iSNVs per host. In contrast, we found a substantial amount of gene content variation: between five and 103 gene gains or losses within each patient. We suggest that most diversity is due to within-host mutation rather than co-infection, and that HGT of mobile elements is more common than point mutations. In most patients, within-host evolution can be explained by neutral mutation, recombination and bottlenecks; however, one patient showed evidence for diversification driven by positive selection, resulting in phenotypic variation among intra-host *V. cholerae* isolates in their ability to form biofilms. Despite the relatively small numbers of mutations and HGT events within hosts, these events may have important evolutionary and phenotypic consequences for *V. cholerae* populations.

## Methods

### Enrolment

To study cholera within-host diversity, stool samples were collected from five patients (B1 to B5) from Dhaka, Bangladesh, and three patients (H1 to H3) from Artibonite, Haiti. Between eight and 20 *V. cholerae* colonies were isolated from each patient, as described below. Patients in Bangladesh were enrolled at the icddr,b (International Center for Diarrheal Disease Research, Bangladesh) Dhaka Hospital. In Haiti, samples were collected from patients presenting to St. Marc's Hospital, Arbonite with acute watery diarrhoea in April 2013. See the Supplementary Methods for more details on sample collection.

In addition to these eight patients, we included 21 ‘Time Course’ patients (TC01 to TC21) from a surveillance program conducted by the icddr,b, between 2011 and 2013. For the Time Course samples, only one isolate was sequenced per patient.

### Sample processing

Stools from both Haiti and Bangladesh were stored at Massachusetts General Hospital (MGH) at −80 °C and then streaked directly onto thiosulfate-citrate-bile salts-sucrose agar (TCBS), a medium selective for *V. cholerae*. After overnight incubation, 20 well-separated colonies were inoculated into 5 ml Luria-Bertani broth and grown at 37 °C overnight. For each colony, 1 ml of broth culture was stored at −80 °C with 30 % glycerol until DNA extraction. For patient 1, one of the colonies was re-streaked on a new TCBS plate and 12 colonies were selected as a control for culture-induced artefacts and sequencing errors. Bacterial stocks made from a single colony were grown in 1.5 ml LB media with agitation at 37 °C for 12 h. Colonies were named with a 'C', followed by a number; for example, B1C1 corresponds to colony 1 from Bangladesh patient 1. All isolates were identified as toxigenic *V. cholerae* O1 biotype El Tor, serotype Ogawa which was the prevailing serotype at each site during the entire study period. Genomic DNA was extracted for each isolate using the Qiagen DNeasy Blood and Tissue kit, using 1.5 ml bacteria grown in LB media. In order to obtain pure gDNA templates, an RNase treatment was followed by a purification with the MoBio PowerClean Pro DNA Clean-Up Kit.

### Whole genome sequencing

Each isolate was separately sequenced using Illumina technology to a minimum depth of 28× coverage of the MJ-1236 reference genome (mean coverage=136×). From 122 isolates retrieved from the eight patients, 66 genomic libraries were constructed using the Nextera DNA library kit (Illumina) according to the manufacturer’s protocol, and were sequenced with the 250-bp paired-end v2 kit on the Illumina MiSeq. The remaining 56 libraries were prepared using the NEBNext Ultra II DNA library prep kit (New England Biolabs) and sequenced on the Illumina HiSeq 2500 (paired-end 125 bp) at the Genome Québec sequencing platform (McGill University). Twelve isolates were sequenced in replicate using both methods. For details about isolates, sequencing and assembly see Table S1.

Each genome was *de novo* assembled, and reads were also mapped to two closed, annotated *V. cholerae* reference genomes. After filtering for errors due to culture and sequencing (see Supplementary Methods), we identified a total of 485 high-quality single-nucleotide variants (hqSNVs) and 22 indels among our 122 isolates, distributed across the genome (Fig. S1), with a majority of hqSNVs (471) located in the branch between the Bangladeshi and the Haitian isolates. Among them, we characterized the intra-host single nucleotide variants (iSNVs) that varied among the isolates taken from a single patient (Table 1).

**Table 1.** Nucleotide and amino acid changes identified in the *V. cholerae* core genome

Region	Type*	Isolates	Ref. nucleotide	Alt. nucleotide	Nucleotide position†	NS/S	Protein	Ref. amino acid	Alt. amino acid	Gene annotation	Patient allele frequency‡
Bangladesh	iSNV	B1C19	C	T	CHR1, 746965	Intergenic	–	–	–	–	1/19
Bangladesh	iSNV	B4C12	C	T	CHR1, 2549743	S	ACQ61457.1	D	D	RNA polymerase sigma-70 factor	1/17
Bangladesh	iSNV	B5C11	T	G	CHR1, 2764922	S	ACQ61649.1	V	V	Toxin co-regulated pilus biosynthesis protein F	1/20
Haiti	iSNV	H1C5	G	T	CHR1, 2240431	NS	ACQ61177.1	R	L	Sensor histidine kinase	1/9
Haiti	iSNV	H1C4 H1C8	G	A	CHR1, 1785021	NS	ACQ60802.1	R	H	TetR family transcriptional regulator	2/9
Haiti	iSNV	H1C6	G	T	CHR1, 2241580	NS	ACQ61177.1	R	L	Sensor histidine kinase	1/9
Bangladesh	Patient	B2+B3	T	C	CHR1, 1295440	Intergenic	–	–	–	–	20/20 20/20
Bangladesh	Patient	B4	C	T	CHR1, 610212	NS	ACQ59736.1	G	R	TatD family hydrolase	17/17
Bangladesh	Patient	B4	C	T	CHR2, 1007076	NS	ACQ62879.1	A	T	Hypothetical protein	17/17
Bangladesh	Patient	B2+B3	G	A	CHR1, 1036008	Intergenic	–	–	–	–	20/20 20/20
Bangladesh	Patient	B4	C	T	CHR1, 1400553	Intergenic	–	–	–	–	17/17
Bangladesh	Patient	B4	G	A	CHR2, 350409	NS	ACQ62264.1	P	L	PTS system mannitol-specific EIICBA component	17/17
Bangladesh	Patient	B2+B3	G	A	CHR1, 2301641	NS	ACQ61224.1	P	S	LacI family transcription regulator	20/20 20/20
Bangladesh	Patient	B1+B5	C	T	CHR1, 359133	N	ACQ59516.1	A	V	Bifunctional purine biosynthesis protein PurH	19/19 20/20

Abbreviations: Ref, reference allele; Alt, alternative allele; NS, non-synonymous; S, synonymous; CHR1, chromosome 1; CHR2, chromosome 2; iSNVs, intra-host single nucleotide variants.

*Mutations segregating within patients are denoted iSNVs; mutations fixed between patients are denoted 'Patient.'

†Nucleotide positions are based on the reference *Vibrio cholerae* MJ-1236 (GenBank accession numbers CP001485.1 and CP001486.1).

‡Patient allele frequency shows the allele frequency of the alternative (minor) allele.

### Estimation of effective population sizes (N_e_)

To estimate effective population size within each patient, we used the formula N_e_=θ/2 µ, where N_e_ is the effective population size, θ is a measure of genetic diversity and µ is the mutation rate [36]. We assumed a mutation rate of µ=1/300 per genome per generation [37]. We report the estimated N_e_ for each patient in Table S2, using both Waterson’s estimator (θ_W_ or S) and Tajima’s estimator (θ_T_ or π) as measures of genetic diversity. We calculate these estimators as described by Tajima [36].

### Characterization of the flexible genome

From assemblies, we annotated the genomes using the RAST pipeline [38] with default parameters. Predicted proteins were used as input for the OrthoFinder software [39] to predict orthologous gene families. These orthologous gene families were classified into three different categories: multiple gene families (>1 copy per genome, on average), single copy genes (exactly one copy per genome) and flexible genomes (<1 copy per genome). Due to variation in sequencing coverage and nucleotide composition, genome assemblies may be incomplete, missing a subset of genes that are actually present [40, 41]. To address this issue, we mapped reads back to the full gene catalogue, and considered a gene to be present when it was covered at 1× (Supplementary Methods; Figs S2 and S3). Potential donors of horizontally transferred genes were identified using blast against NCBI GenBank, followed by phylogenetic analyses (Supplementary Methods).

### Phylogenetic evolutionary inference and root-to-tip regression

For all phylogenetic analyses, we excluded the Integrative Conjugative Element (ICE), a highly variable 100-kb region (Fig. S1) that undergoes frequent HGT and thus has a separate evolutionary history from the rest of the core genome [6]. The ICE was defined as the region of MJ1236 chromosome 1 located between positions 87 776 and 193 789 (after reverse-complementation of MJ1236), according to a previous study [42]. A final alignment of 201 concatenated hqSNVs was generated from the core genome of the 35 genotypes (TC01–TC21 plus one to four unique genotypes per patient) and used for phylogenetic analysis. Seaview v.4.5.4 [43] was used to generate a maximum-likelihood phylogeny, employing a general time reversible (GTR) substitution model with four rate classes and subtree pruning and regrafting (SPR) branch-swapping. All sites being variable in the alignment, we did not consider the proportion of invariable sites. To test the degree of temporal structure of our data we performed a root-to-tip linear regression using the TempEst software [44], which suggested that our dataset is sufficiently clock-like to robustly estimate an evolutionary rate (R^2^=0.65, *P*<0.0001; Fig. S4). Substitution rates and divergence times were then estimated using beast v.1.8.3 [45], with XML-input files manually modified to include both variable and invariable sites, for a total genome the size of the *V. cholerae* MJ-1236 reference. The Bayesian tree was computed and calibrated with sampling dates of the isolates ranging from March 2011 to December 2013. We compared different molecular clock and demographic models (Supplementary Methods), and found that the strict molecular clock and the Bayesian skyline plot models provided the best fit (Table S3), in accordance with previous studies of *V. cholerae* [12, 46].

### Tests for violations of neutral evolution

We conducted permutations of the distribution of non-synonymous, synonymous and intergenic SNVs across sites in the genome and branches of the phylogeny to identify any possible deviations from neutral evolution (Supplementary Methods). To investigate the role of natural selection within versus between patients, we performed the McDonald–Kreitman test [47] and also conducted permutations to identify any patients with an excess of non-synonymous iSNVs compared to random distribution of iSNVs across patients (Supplementary Methods).

### Liquid culture and biofilm growth assays of isolates from patients H1 and H2

To identify possible phenotypes conferred by the within-patient variations, we performed *in vitro* experiments on the isolates from patient H1 harbouring the three NS point mutations, and the isolate for which the *Bacteroides* plasmid was detected in patient H2. First, to test whether intra-host variants had any effect on *V. cholerae* growth in liquid medium, we measured the growth rates of the variable isolates compared to one isogenic isolate (with inferred ancestral alleles and no variation in the flexible genome) from the same patient. Isolates were grown in 4 ml LB broth with agitation at 30 °C, and optical densities were measured at 600 nm using a spectrophotometer every hour for 12 h. Second, to test for biofilm production, we grew the same isolates in 200 µl LB in a 96-well plate, without agitation at 30 °C for 48 h. Controls included empty wells, wells with LB only, and wells with a *V. cholerae* isolate with an in-frame deletion in the gene *vpsA* (Vibrio polysaccharide A), which results in reduction of biofilm production [48]. A 0.1 % solution of crystal violet was used to stain for biofilm adherent to the well. Biofilms were dissolved in ethanol at the end of the assay, and the optical density was measured at 595 nm using spectrophotometry. Experiments were performed in replicates of four to 12.

### Polymyxin B MIC assay on patient H1 isolates

Isolates H1C1, H1C5 and H1C6 were grown overnight at 30 °C on LB agar and then cultures were diluted 1 : 100 in fresh LB medium. Cells were grown to mid-exponential growth and diluted 1 : 10, and an aliquot was plated on LB agar. Polymyxin B E-test gradient strips (AB Biodisk) were applied to inoculated plates and incubated at 37 °C, and the minimum inhibitory concentrations (MICs) were evaluated after 16 h.

## Results

### Within-patient single nucleotide variation

**Fig. 1.**
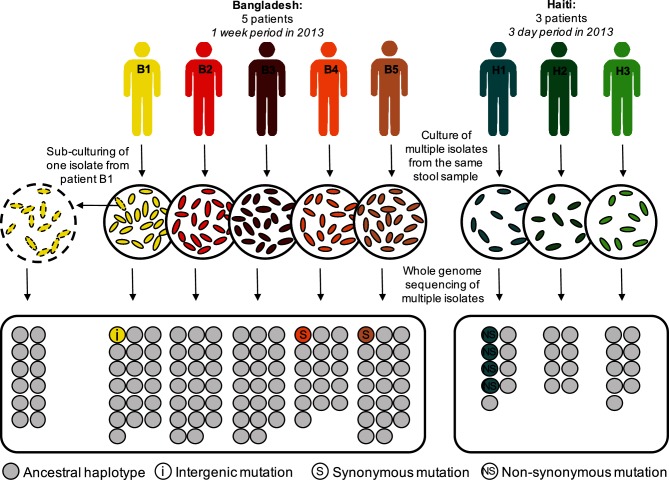
Culture and sequencing of isolates of *Vibrio cholerae* from eight acutely infected patients. To study within-patient evolution, selective media was used to culture stool samples from five patients from Bangladesh (B1 to B5) and three patients from Haiti (H1 to H3). Between eight and 20 colonies were isolated from each patient and sequenced separately. For patient B1, we performed a sub-culture of one isolate (dotted outline) and sequenced 12 of these new isolates as a control for cultured-induced and sequencing artefacts. We independently called variants, compared them between isolates within each patient to identify the intra single nucleotide variants (iSNVs, coloured circles) and determined whether they were intergenic (i), synonymous (S), or non-synonymous (NS) mutations.

Among the 485 hqSNVs detected, we found intra-host single nucleotide variants (iSNVs) in patients B1, B4, B5 and H1, from whom we sequenced respectively 19, 17, 20 and 9 isolates (Fig. 1). Isolates from patients B2, B3, H2 and H3 (20, 20, 8 and 9 isolates, respectively) were all isogenic within patients, with no iSNVs detected using our quality filters. Patient B1 contained one intergenic iSNV (with an allele frequency of 1/19), and patients B4 and B5 each contained a synonymous iSNV, each in a different gene (with respective allele frequencies of 1/17 and 1/20) (Table 1). Patient H1 contained three iSNV sites, all of which were non-synonymous, and two of which occurred in the same gene, a sensor histidine kinase (Table 1), and one of which was at frequency 2/9, for a total of 4/9 isolates containing iSNVs (Fig. 1). Twenty-two small insertion/deletions (indels) were found to vary between patients (and specifically between patients sampled in different years or different countries), but we did not detect any indels that varied within patients.

To ensure that the relatively small number of iSNVs were not due to mutation during isolate isolation and culture [49] or sequencing errors, we sub-cultured and sequenced 12 colonies from one isolate (B1C1) as a control (Fig. 1). Applying the same filters as for SNV discovery in our patients, we did not detect any iSNVs among control isolates, nor did we detect any SNV differences between replicate libraries prepared and sequenced using different platforms (Methods). This suggests that the few iSNVs identified within patients are unlikely to be culture or sequencing artefacts.

Based on the number (0–3) and frequencies of iSNVs per host, we used measures of genetic diversity (θ_W_ and π) to estimate within-host effective population size (N_e_). We estimated that N_e_ within each patient ranged from 0 to 110 (Table S2). Such a small N_e_ in a bacterial population is consistent with a recent population bottleneck, possibly during host colonization, or a recent selective sweep having purged most of the diversity within the population.

### Gene gain and loss within and between cholera patients

**Fig. 2.**
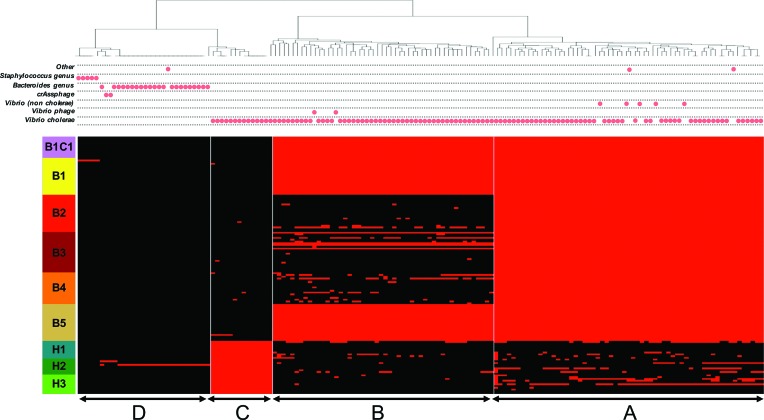
Presence/absence profile and taxonomic affiliation of gene families in the flexible genome. Red in the heatmap indicates gene presence; black indicates absence. Each column shows the presence/absence profile for a unique gene family. The heatmap is ordered by patient along the vertical axis. B1C1 is the control, subcultured from patient B1, and contains no flexible genome variation. The horizontal axis is ordered by hierarchical clustering, yielding four clusters: A, B, C and D. The taxonomic affiliation of each gene family (best blast hit) is indicated with dots above the heatmap.

To characterize variation in gene content among the 122 sequenced isolates, we analysed orthologous coding sequences from *de novo* assemblies. We defined the core genome as the genes present in all isolates, and the flexible genome as the genes absent in some of the isolates. We defined a flexible genome of 155 genes that varied in their presence or absence across genomes (Methods). Some of these flexible genes varied in presence/absence within patients, ranging from five to 103 genes depending on the patient considered (Table 2, Fig. 2). As two different methods of library construction and sequencing were used in this study, we sequenced twelve isolates using both methods. Among them, we observed variation in the detection of the flexible genome for six of the duplicate sets (Fig. S5), likely because different library preparation methods may have different G+C content biases [50]. Using genomes from one method only (NEBNext/HiSeq), we identified between five and 67 variable genes per patient; using the other method (Nextera/MiSeq) we identified between zero and 62 genes (Table S4). In six out of the eight patients studied, the NEBNext/HiSeq prep identified more within-patient variable genes than the Nextera/MiSeq prep. However, for the other two patients, the Nextera/MiSeq prep detected more variable genes; we therefore hesitate to draw general conclusions on which method performed best. When a variable gene is not detected in a given method, we consider this a false-negative. We conclude that methodological differences alone cannot explain the flexible genome variation within patients, and consider both methods combined for the remainder of the paper.

**Table 2.** Flexible gene content variation within and between patients

Patient	No. genes fixed within patients	No. genes variable within patients	No. singletons
B1	111	11	5
B2	61	35	0
B3	61	51	0
B4	61	49	0
B5	111	5	0
H1	14	68	0
H2	14	103	25
H3	14	63	0

Singletons are defined as genes only found in one isolate, and are also counted as variable genes within patients. Genes fixed within patients are present in all isolates from a patient, but are absent in at least one other isolate in the study.

Clustering the 155 flexible genes by their presence/absence profile across patients revealed four distinct categories of genes (Fig. 2). Category A consists of genes present in Bangladesh (part of the Bangladesh core genome), but showing a patchy distribution in Haiti. Category B genes are fixed (present in all isolates) in patients B1 and B5, and patchy in other patients. Category C genes are fixed in Haiti and nearly absent from Bangladesh. Category D genes tend to be rare, often singletons only observed in a single isolate within either patient B1 or patient H2.

Several flexible genes corresponded to known mobile genetic elements. Notably, category A contains 61 gene families (39 % of the flexible genome), all located on one single contig (possibly gained/lost in a single event) corresponding to a SXT Integrative Conjugative Element (ICE). Category B encompassed 49 gene families matching Kappa phage proteins. These putative phage genes were clustered together on large contigs of chromosome 1, and were fixed in patients B1 and B5 but variable among other patients, some of which contained complete phage sequences (patients B2 and B3). Category C contained 15 genes, including some present in the ICE, which mapped to at least five different contigs (depending on which isolate's assembly was considered), suggesting multiple gain/loss events or frequent rearrangements.

Over half of the flexible genome (80 genes) was annotated as hypothetical proteins, compared to the core genome which contained less than 3 % hypothetical proteins. The flexible genome also contained 10 transposases (6.5 % of the flexible genome, compared to 1.4 % of the core) and eight genes involved in plasmid and viral replication, all potential mechanisms of HGT [51]. A complete list and annotation of flexible genes is given in Table S5.

Variation in the flexible gene pool could arise from gene deletion, duplication, or HGT. To determine the extent of HGT across species boundaries, we identified the taxonomic affiliation of each flexible gene according its placement on a phylogeny of homologs from the GenBank database (Methods). While the majority (117 out of 155) of flexible genes were assigned to *V. cholerae,* several were assigned to non-cholera vibrios or even distantly related species of *Bacteroides* or *Staphylococcus* (Fig. 2). These genes had no blast hits to *Vibrio*, but numerous hits to *Bacteroides* or *Staphylococcus,* suggesting HGT from these donors to *V. cholerae* in the gut. For example, a group of 20 genes present in isolate H2C3 (but absent in other isolates from patient H2) matched a plasmid previously identified in *Bacteroides* (Fig. 2). These 20 genes of putative *Bacteroides* sp. plasmid origin are among 25 singletons, present in only one isolate of patient H2. Similarly, the five singletons in patient B1 (Table 2) are all of putative *Staphylococcus* origin. Each of these 25 genes are located on a different contig where a single gene is predicted, except for two *Bacteroides* sp. genes, identified on the same contig. We are therefore unable to conclude whether the genes are integrated into a *V. cholerae* chromosome or as part of a plasmid. Aside from the genes suspected to have been transferred, no other reads from genomes of *V. cholerae* isolates mapped to *Staphylococcus* or *Bacteroides*, suggesting that putative HGT events were not due to contamination. Together, these results suggest that most within-patient variation in gene content is due to gene flow, deletion or duplication within the *V. cholerae* population, with rare but detectable HGT from other bacterial species, phages and plasmids in the gut microbiota. Owing to their low frequencies within patients, these cross-species HGTs are likely rare and recent events, which may never achieve high frequency in the *V. cholerae* population, either within or across hosts.

### *V. cholerae* evolution on different time scales

**Fig. 3.**
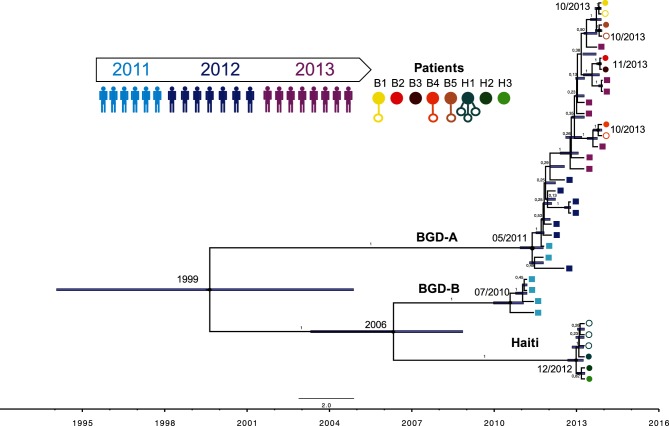
Bayesian phylogenic tree of 35 *V. cholerae* genotypes sampled over 3 years in Bangladesh and Haiti. The maximum clade credibility tree represents the genealogy of sequences in the study, reconstructed from concatenated hqSNVs, using beast. Coloured squares (shades of blue and purple) represent the time-course isolates collected from Bangladeshi patients from March 2011 to December 2013 (one isolate per patient). Patients for whom we measured intra-host variation (B1–B5 and H1–H3) are shown as circles. Filled circles indicate the putative ancestral genotype, and empty circles indicate putatively derived iSNVs. The median node age and divergence date in months and years are indicated at the nodes. The blue bars represent the 95 % HPD intervals for divergence time estimates, and posterior probabilities are represented on the branches.

In order to place within-host variation in the context of longer-term *V. cholerae* evolution, and to distinguish within-patient mutation from co-infection events, we built a phylogeny of the 122 isolates (all from 2013) as well as 21 additional isolates obtained from acute cholera patients sampled in Bangladesh from 2011 to 2013 (Fig. 3). Assuming a constant evolutionary rate across lineages, we estimated the evolutionary rate at 7.94×10^−7^ hqSNV site^−1^ year^−1^ [95 % highest posterior density (HPD), 4.89×10^−7^ to 1.14×10^−6^] or approximately 3.3 hqSNV year^−1^ in the core genome (95 % HPD), consistent with previous estimates [6].

We then estimated the ages of the most recent common ancestors (MRCAs) of the phylogenetic sub-lineages and clusters (Table S6). Notably, four of the six isolates from 2011 in Bangladesh were found to be closer to the Haitian isolates than to all the other Bangladeshi isolates (Fig. 3). We called this sub-lineage BDG-B, and estimated the age of the MRCA of these four isolates and the Haitian isolates in September 2005 (95 % HPD, June 2002 to July 2008), which pre-dates the introduction of pathogenic *V. cholerae* in Haiti, and is consistent with its Asian origin [10, 11]. The time of the MRCA of the isolates collected from the three Haitian patients was estimated at December 2012 (95 % HPD, August 2012 to March 2013).

Based on the phylogeny, we sought to distinguish between scenarios of within-patient mutation or co-infection as causes of within-patient diversity. It is clear that isolates from the same patient always grouped together, and were never polyphyletic (Fig. 3). This observation is consistent with each patient being colonized by a single clone, which subsequently diversified by mutation within the patient. The diversity between the eight patients (7 SNVs) was greater than the diversity within patients (0–3 iSNVs), which would be unlikely if cells of *V. cholerae* were sampled by patients at random from an environmental pool (Table 1). If within-patient diversity was due to co-infection of the same patient by multiple different strains, we would expect these strains to share a MRCA before the date of infection, and certainly before the date of stool sampling. However, the MRCA of isolates from a single patient always overlapped with the date of sampling, suggesting that within-patient diversity is more likely due to within-patient mutation than to co-infection.

### Signatures of natural selection on within-patient variants

Over 3 years of evolution, we could not reject a neutral evolutionary model and found no evidence for variation in the NS:S ratio over time, considering only SNVs fixed between patients (Supplementary Note; Fig. S7). Another possibility is that selection acts over shorter evolutionary scales, by shaping intra-host diversity during acute infection. Under this scenario, we would expect NS:S ratios to differ significantly within and between hosts. For example, higher NS:S within than between hosts could be due to positive or balancing selection on NS mutations within hosts, or due to more efficient purifying selection (against deleterious NS mutations) between hosts. To test for such deviations from neutral evolution, we applied the McDonald–Kreitman test [47] to the eight hosts surveyed for within-host genetic variation (five from Bangladesh and three from Haiti). Despite the overall low number of SNVs and iSNVs observed, we found a significant excess of NS mutations between Bangladeshi patients (Fisher’s exact test, Odds Ratio >12, *P*<0.05; Table 3), suggesting positive selection for the fixation of NS mutations between patients, or purifying selection against NS mutations within patients. In contrast, all three iSNVs observed in Haiti were NS, suggesting positive, balancing, or relaxed purifying selection within patients, although not statistically significant (Fisher's exact test, Odds Ratio <0.32, *P*=1; Table 2).

**Table 3.** McDonald–Kreitman test for differential selection within and between patients

	Bangladesh		Haiti	
Population	NS	S	NS	S
Polymorphic (within patient)	0	2	3	0
Fixed (between patients)	5	0	0	0
	*Fisher exact test, P=0.048*		*Fisher exact test, P=1*	

Counts of non-synonymous (NS) and synonymous (S) polymorphic sites (within patient iSNVs) and fixed sites (between patients) for Bangladeshi and Haitian patients.

**Fig. 4.**
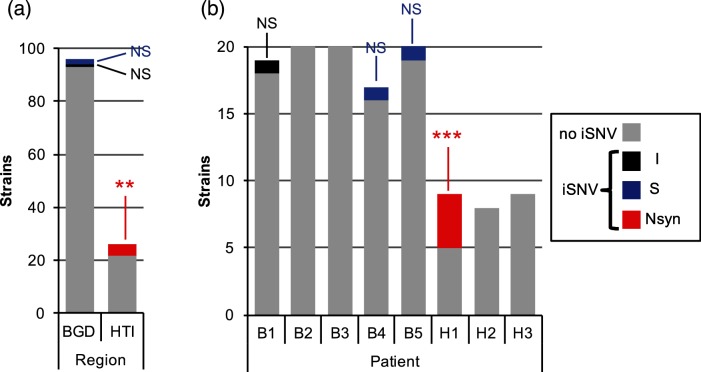
Significant excess of non-synonymous iSNVs in patient H1. (a) Distribution of 122 *V. cholerae* isolates containing different categories of iSNVs (I, intergenic; S, synonymous; Nsyn, non-synonymous) or no detectable iSNVs, according to geographic region (BGD, Bangladesh; HTI, Haiti). Patients from Haiti have a significant excess of Nsyn iSNVs (red; **P≤0.01; 10 000 random permutations of isolates among regions). (b) Distribution of 122 *V. cholerae* isolates containing different iSNVs, or no detectable iSNVs, by patient. Patient H1 has a significant excess of isolates with Nsyn iSNVs (**P≤0.001; 10 000 random permutations of mutations across patients; Supplementary Methods). ns, not significant.

The three NS iSNVs observed in Haiti all occurred within a single patient, possibly driven by selective pressures specific to this patient (Table 1). To test whether this pattern of iSNVs was likely to have occurred at random or due to patient-specific selection, we performed permutations of iSNVs among hosts and estimated expected iSNVs frequencies (*F*) and number of NS iSNVs per host and region. We found a significant excess of iSNVs in Haiti (*F*_HTI_=0.15; *P*<0.05; 10 000 permutations) and in patient H1 (*F*_H1_=0.44; *P*<0.01), but not in Bangladesh (*F*_BGD_=0.03; *P*>0.05) nor in any other patients (*F*=0–0.05; *P*>0.05). All iSNVs identified in Haiti and in patient H1 were non-synonymous, which was significantly higher than expected by chance (*P*<0.01 and *P*<0.001, respectively; Fig. 4). These results show that patient H1 has a significant excess of NS iSNVs compared to other patients. This suggests positive or balancing selection on NS iSNVs within patient H1, or relaxed purifying selection in patient H1 compared to other patients.

The three NS iSNVs in patient H1 occurred in two genes. The first gene, containing one iSNV at position 1 785 021 on chromosome 1 of the MJ-1236 reference genome (Table 1) encodes a member of the tetracycline resistance (Tet^R^) family of transcriptional regulators (NCBI accession number ACQ60802.1), known to be involved in the transcriptional control of multidrug efflux pumps and other pathways like quorum-sensing circuits or pathogenicity [46, 52]. The other two NS mutations (positions 2 240 431 and 2 241 580 of chromosome 1) in patient H1 were located in the same gene (NCBI accession number ACQ61177), a sensor histidine kinase (HK) called *vprB*, which is required for resistance to the antimicrobial peptide polymyxin B [53]. Each of these two iSNVs occurs at a different site in the gene, each in a different isolate. Based on the fact that the major allele at each of these iSNV sites was present in both reference genomes (MJ1236 and 2010EL-1786), we inferred that the minor alleles (both at frequency 1/9 in patient H1; Table 1) were derived, presumably due to within-patient mutation. A comparison of the *vprB* (ACQ61177) protein sequence with its 465 closest orthologs revealed that the NS iSNVs modify peptides that are otherwise highly conserved across species of the genus *Vibrio* (Fig. S6), suggesting that these mutations may affect protein function.

### Within-patient variants affect biofilm formation

We next asked whether any of the intra-host variants affected *V. cholerae* phenotypes. We focused on the non-synonymous SNVs in *vprB* which showed a signature of positive selection in patient H1, and on the plasmid of putative *Bacteroides* origin that varied in presence/absence within patient H2. First, we established that *V. cholerae* isolates (with or without plasmid, or with ancestral or derived *vprB* alleles) did not differ in growth rate in rich medium (Methods). As loss of *vprB* function has been previously associated with increased susceptibility to polymyxin B [53], we also tested for resistance to the antibiotic polymyxin B, and again found no difference between isolates. Therefore, we chose to focus on biofilm formation, a trait which can impair intestinal colonization but might be beneficial in the aquatic environment [54, 55], and is readily quantifiable. Furthermore, it is known that HKs in certain two-component systems can affect biofilm formation [56, 57] but the role of the *vprB* HK in particular is unknown.

**Fig. 5.**
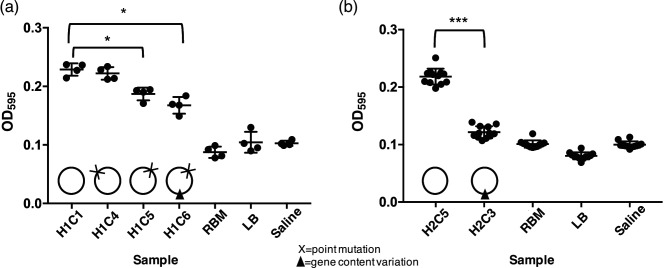
Biofilm formation of isolates from patients H1 and H2. Optical density at 595 nm was measured for four to 12 replicates of each isolate, after growth for 48 h at 30 °C. Statistical comparisons were made using a non-parametric Mann-Whitney test (*, *P*<0.05; ***, *P*<0.0001). Circles represent genomes with either variation in gene content (dark triangle) or iSNV variation (cross). (a) Isolates from patient H1. Isolate H1C1 represents the ancestral genotype, H1C4 has a non-synonymous mutation in a transcriptional regulator gene, and H1C5 and H1C6 have different non-synonymous mutations in the same gene, the histidine kinase gene. (b) Isolates from patient H2. Isolate H2C5 represents the ancestral genotype, with no variation in the gene content, and H2C3 harbours a plasmid. RBM is a biofilm knockout strain, and LB and saline are negative controls.

We found that H1C5 and H1C6, the two isolates with derived alleles in the HK gene *vprB*, produced significantly less biofilm than the other isolates from the same patient (Fig. 5a). Based on our hqSNV calls and flexible gene analysis, the genome of H1C5 was identical to H1C1, with the exception of the iSNV in the HK gene. Therefore, the difference in biofilm phenotypes is attributable to this iSNV. However, H1C6 differed from H1C1 by an iSNV in the HK gene, and also by the presence/absence of genes in the flexible genome (Fig. S5). However, this gene content variation did not measurably affect biofilm formation (Fig. 5a). In contrast to the differences in biofilm formation between *vprB* alleles, we did not detect any significant difference in biofilm formation between isolates from patient H1 with the ancestral iSNV allele (in isolate H1C1) or the derived allele in the transcriptional regulator gene (isolate H1C4). In summary, within-patient mutations in *vprB*, but not another mutation within the same patient, significantly reduced the ability of *V. cholerae* to form biofilms.

In the case of patient H2, we found that the presence of a plasmid of putative *Bacteroides* origin reduces biofilm formation even more strongly than point mutations in *vprB*. Specifically, the *Bacteroides* sp. plasmid-containing isolate (H2C3) produces approximately two-fold less biofilm than an isogenic control from the same patient (H2C5). The biofilm formation of H2C3 was indistinguishable from negative controls (Fig. 5b). Together, these results show that both point mutations and plasmids segregating within patients can affect biofilm formation.

## Discussion

In this study, we surveyed the genetic diversity of *Vibrio cholerae* within infected patients. Using whole-genome sequencing, we analysed 122 clinical isolates from eight cholera patients from Bangladesh and Haiti, and demonstrated that overall levels of within-patient variation are low for *V. cholerae* populations compared to more chronic bacterial pathogens, which routinely harbour more than 20 iSNVs per patient [25–29, 58]. Even if rare, point mutations may be under selection within hosts, with phenotypic consequences. For example, we showed that intra-host mutations in a sensor histidine kinase gene reduced biofilm formation. In addition to point mutations, HGT plays a major role in *Vibrio cholerae* evolution and may represent the major source of genetic diversity, not only in the aquatic environment, but also in the human host – and with large effects on phenotypes like biofilm formation. Specifically, different mutations in a sensor histidine kinase and the acquisition of a *Bacteroides* sp. plasmid both reduced the ability of *V. cholerae* to form biofilms, which could be advantageous during host colonization [53, 54].

### Gene content variation within patients and its functional consequences

While HGT is already well-characterized on longer epidemiological time-scales, we show that it also occurs within individual patients. *V. cholerae* is known to undergo HGT via transformation [59], transduction [60] and conjugation [61, 62]. HGT contributes substantially to drug resistance, pathogenicity and adaptation to different environments, via the acquisition of genomic islands, phages, transposons, ICEs and plasmids [7, 63]. Our characterization of the flexible genome within patients used read mapping to confirm gene absences, reducing false-positive inference of gene content variation (Figs S2 and S3). We detected between five and 103 genes that varied in presence/absence within patients (Fig. 2; Table 2). Each gene does not necessarily represent an independent gain/loss event; for example, the ICE contained 61 genes on a single contig, likely a single gain/loss event. Even under the conservative assumption that all gene content variation represents a single gain/loss event per patient, this still indicates at least one event per patient.

Some of the putative HGT events could have consequences for *V. cholerae* survival and virulence within the host. For instance, the group of 20 genes acquired by one *V. cholerae* isolate within patient H2, likely via a plasmid of *Bacteroides* origin, is associated with a twofold reduction in biofilm formation (Fig. 5). Among these 20 genes, we identified an antibiotic resistance gene, a haloacid dehalogenase protein that could impact pathogenicity [64], and a FtsY recognition signal protein that was shown to increase virulence in *Streptococcus* [65]. Among the genes likely acquired from a *Staphylococcus* donor in isolate B1C2, three could potentially be involved in modulation of virulence (Fig. S5, Table S6). Firstly, a GNAT family acetyltransferase could promote virulence or increase antibiotic resistance [66, 67]. Secondly, a putative phosphoenolpyruvate phosphotransferase has been demonstrated to play a role in biofilm formation in *Vibrio* [68–70]. Finally, the KdpC gene (a potassium-transporting ATPase) has been shown to modulate virulence in *Mycobacterium paratuberculosis* [71].

Not all gene gain/losses are due to HGT. Many can be explained by gene deletions, such as phage excision events. Deletions could explain much of the variation among genes in categories A and B (Fig. 2), respectively corresponding to the ICE and Kappa phage. These elements are known to vary among *V. cholerae* genomes sampled over larger temporal and geographic scales [72], but here we document likely excision events during human infection. Genes in category D tend to be singletons, present in just a single isolate, and with taxonomic affiliations well beyond *V. cholerae*, including *Bacteroides, Staphylococcus*, and crAssphage (Fig. 2). Category D genes are most easily explained by cross-species HGT, as previously documented by Folster and colleagues, who identified a Haitian *V. cholerae* isolate that gained multidrug resistance through transfer of a plasmid from a species of *Enterobacteriaceae* [61]. Although to our knowledge, ours is the first description of HGT specifically between *V. cholerae* and *Bacteroides*, the genus *Bacteroides* is known to be involved in inter-species and inter-genus HGT in the human gut [73, 74]. Taken together, these results are consistent with the human gut being a hotspot of HGT [75], sometimes involving pathogens like *V. cholerae.* Although we hesitate to speculate on the eventual fate of within-patient gene gain/loss events, it appears that certain events (e.g. in the ICE or kappa phage) persist long enough to be observed as fixed differences between patients. The rare cross-species HGT events we observed were at low frequency (present in just one isolate per patient), suggesting they are neutral or slightly deleterious variants that will never attain high frequency – although their eventual fate is unknown.

### Regimes of natural selection inferred from within-patient point mutations

The low levels of variation (0–3 iSNVs per patient) observed within cholera infections could be easily confounded with sequencing errors or mutations occurring during culture rather than within patients. Therefore, we developed filters for calling SNVs and gene gain/loss events that yielded zero variation among control isolates, suggesting low rates of false-positive variant calls and increasing confidence that the six total iSNVs (Fig. 1; Table 1) did indeed vary within patients.

Point mutations detected within cholera patients could be the result of *de novo* mutations occurring within the patient, or a consequence of a co-infection from different strains that had diverged previous to the infection. Although we cannot formally exclude co-infections (particularly of low-frequency strains not detectable by sequencing 8–20 isolates), our results are more consistent with *de novo* mutation within hosts. Isolates from the same patient were grouped together on the phylogeny (Fig. 3), suggesting a recent clonal ancestor. This result is consistent with previous findings that cholera outbreaks are highly clonal [76].

Cycles of transmission from host to host, or from aquatic environment to host, can induce population bottlenecks, reducing the effective population size. Based on comparisons of distantly related genomes, N_e_ of *V. cholerae* has been estimated to be 4.78×10^8^ [77]. This relatively large N_e_ reflects the high genetic diversity present in the aquatic environment, and over long evolutionary time-scales. However, during transmission and intestinal colonization, the size of the *V. cholerae* population experiences drastic bottlenecks that could temporarily reduce N_e_. Abel and colleagues showed that *V. cholerae* population sizes in rabbit models of infection ranged from 10^5^ during the early phases of colonization to ~10^2^ at the late phases of infection [32]. Our estimates of N_e_ based on iSNVs give values of 0 to ~10^2^, consistent with population bottlenecks or selective sweeps purging diversity (Table S1). The infectious dose of *V. cholerae* has been estimated to be 10^3^–10^8^ cells [1]. Our low estimates of N_e_ indicate that (i) this infectious dose is genetically homogeneous, or that (ii) any pre-existing diversity is quickly purged by a bottleneck or selective sweep.

In addition to the small within-patient N_e_, we found that the distribution of mutations, physically along the *V. cholerae* genome, and temporally along the phylogeny, could generally be explained by random neutral simulations (Supplementary Note). However, we identified an excess of NS mutations in one Haitian patient (H1), suggesting positive or diversifying selection on *V. cholerae* within this patient (Fig. 4). Patient H1, like all patients in this study, suffered from severe acute cholera, and we do not have access to further information about this patient which might explain the excess of NS mutations. Two of these mutations affected the same protein, a sensor protein histidine kinase. This sensor protein histidine kinase (HK) is part of a two-component system known as VprAB, which has been shown to mediate glycine fixation in the lipid A domain of lipopolysaccharide molecules, which is necessary for resistance to the antimicrobial peptide polymyxin B [53]. Another two-component system, CarRS, is known to confer polymyxin B resistance, but also to negatively regulate biofilm formation [56, 57]. Here, we found that derived alleles (presumed *de novo* mutations within the host) in the HK VprB did not appear to affect polymyxin B resistance, but did reduce the ability of *V. cholerae* to form biofilms (Fig. 5). It has been previously suggested that biofilm formation may be beneficial for survival in the aquatic environment, but detrimental to survival or colonization of mammalian hosts [54, 55]. Therefore, the derived iSNV alleles may have been selected for reduced biofilm formation.

Our study provides an opportunity to compare *V. cholerae* evolutionary dynamics between Bangladesh, where cholera has been endemic for hundreds or thousands of years [78], and Haiti, where it was introduced in 2010. Our results suggest that selective pressures on *V. cholerae* may differ between Haiti and Bangladesh, as previously proposed [12]. In Bangladesh, we observed an excess of NS mutations between patients (Table 3), suggest positive selection on protein sequences between patients, or efficient purifying selection purging NS mutations within patients. In contrast to Bangladesh, where zero NS iSNVs were observed, we observed three NS iSNVs in Haiti, all within the same patient. Such differences between Haiti and Bangladesh need confirmation in a larger sample, and if confirmed could have different explanations. For example, if Haitian patients are less likely than Bangladeshis to have had prior exposure and immunity to cholera, perhaps cholera infections could last longer, or support larger *V. cholerae* population sizes within patients in Haiti, allowing more efficient positive selection within patients. Further sequencing of intra-host *V. cholerae* genomes, ideally in combination with clinical data on infection durations and outcomes, will be needed to test this hypothesis.

### Conclusion

We have shown that small but measurable changes occur in the *V. cholerae* genome during human infection. Changes in flexible gene content appear to accumulate more quickly than point mutations, although point mutations may also be targets of natural selection. Both gene content variation and point mutations can have consequences for the phenotypes of within-patient *V. cholerae* populations, including clinically- and environmentally-relevant traits like biofilm formation. Future studies will be necessary to determine the role of intra-host diversity – particularly in the ICE and mobile genetic elements – in the evolution of antibiotic resistance, host adaptation, and the severity of disease in infected patients.

## Data bibliography

1. Levade *et al.* Sequence Reads Archive, SRP116359 (2017).
2. Levade *et al*. Github. 2017. https://github.com/ilevade/Vibrio_cholerae_within_patient_assemblies.

## Supplementary Data

1310 Supplementary File 1

## Supplementary Data

52 Supplementary File 2

## Supplementary Data

53 Supplementary File 3
