# Supplementary material for: Vibrio cholerae genomic diversity within and between patients
Source: Microb Genom. 2017 Dec 7;3(12):e000142. doi: 10.1099/mgen.0.000142 (PMC5761273; doi:10.1099/mgen.0.000142)
Supplement: Supplementary File 1 [file mgen-3-142-s001.pdf]

Supplementary Methods

Enrollment and sample processing.

The icddr,b cares for more than 120,000 patients annually including approximately 20,000 with cholera. Patients presenting during 2013 with acute watery diarrhea were eligible for inclusion in this study if stool cultures were positive for *V. cholerae* as the only pathogen, if they were between 2 and 60 years of age, resided in or around Dhaka, and were without major comorbid conditions. Diarrheal samples were then examined by dark field microscopy and if positive for *V. cholerae* on presentation, then stool was cultured overnight. Samples with visible *V. cholerae* growth were serologically confirmed by slide agglutination with specific monoclonal antibodies for Ogawa or Inaba serotypes [1]. Confirmed cholera stool was stored in glycerol at -80°C and shipped to Massachusetts General Hospital, Boston. In Haiti, fresh stool from suspected cholera patients was stored in glycerol at -80°C and shipped from St. Marc's Hospital to Massachusetts General Hospital, Boston.

Genome assembly

To exclude low-quality sequences, we filtered raw reads with Trimmomatic [2]. The 15 first bases of each read were trimmed and reads containing at least one base with a quality score of <30 were removed. *De novo* assembly was then performed for each isolate using IDBA_ud v1.1.1[3].

Filtered reads were also mapped with Bowtie2 v2.2.5[4] to a total of 11 references: two annotated reference genomes, one from Haiti (2010EL-1786, accession

no. NC_016445.1 and NC_016446.1), one from Bangladesh (MJ1236, accession number NC_012667 and NC_012668), and nine assembled genomes (one from each patient and one from the sub-cultured control colonies: B1C1-06, B1C7, B2C12, B3C12, B4C5, B5C10, H1C5, H2C3, H3C2). PCR duplicates were removed from mapping using the MarkDuplicates function of PICARD TOOLS v1.130 (https://broadinstitute.github.io/picard/) and the SAMtools view utility [5], and realignment around indels was performed using the Genome Analysis Toolkit v3.1.1, with default settings. To facilitate the identification of homologous regions among the eleven reference genomes, MJ1236 and the nine *de novo* assembled genomes were aligned against the 2010EL-1736 genome using the "move contig" option in Mauve v. 2.4.0 [6], with default parameters.

Variant calling and annotation

We used SAMtools v1.3 and BCFtools v1.1 to call SNVs and indels from mapping, requiring a minimum mapping quality of 30 and a minimum base quality of 20. Resulting SNVs and indels were then filtered by quality score (<20), depth of coverage (<10) and FQ scores (<0, lower values indicate agreement between reads) with VCFlib [7]. For each isolate, we also filtered variable positions that were not retrieved in all the mappings against all 11 references, by performing reciprocal BLAST of the 50 nucleotides upstream and downstream of each variable position and comparing them. Only matches with >95% identity were kept and multiple matches were excluded as possible duplications and repeated elements. After applying these filters, we compared the genomes of the 12 replicate clones from the isolate B1C1, to control for possible SNVs due to mutations during culture, or sequencing errors. We also removed positions

that were called as variable when reads from one isolate were mapped to the assembly from the same isolate. We considered these SNVs as potential sequencing, mapping or assembly errors. Using these filters, we generated a list of high-quality SNVs (hqSNVs). From this list, we identified intra-host single nucleotide variants (iSNVs) as SNVs that were polymorphic among isolates from the same patient. No iSNVs were identified among the control colonies (the 12 replicate clones subcultured from B1C1).

Annotations were available for the MJ1236 reference genome and were retrieved from GenBank files (Chromosome 1: CP001485.1; chromosome 2: CP001486.1). Variants from the core genome were classified in three categories: intergenic (INT) when falling outside of a coding region, synonymous (S) when affecting the nucleotide sequence of at least one gene but not its amino-acid sequence; or non-synonymous (NS) when affecting the amino acid sequence of at least one gene.

Characterization of the flexible and core genomes

Of 3907 gene families identified, 3489 were defined as core (i.e. present in all genomes) while 401 (~10% of the total gene pool) were considered flexible, present in only a subset of genomes. As absence of a given gene in a genome could be an artifact of the assembly process, we confirmed the absence of each gene family using the raw reads (Fig. S2). A representative catalogue of the flexible genome protein sequences was built using the cd-hit program with a 90% similarity threshold [8]. We used sequences of the catalogue as queries for a blastn search on raw reads. We considered a gene family to be present in a given genome if the average coverage of the gene was greater or equal to 1X. This coverage threshold allowed us to detect every single gene in the gene catalogue (Fig. S3) while observing no variability among the control isolates.

To calculate coverage, we summed the length of all reads matching a given query over a minimal length of 100 nucleotides and a minimal identity of 97%, and divided by the gene length.

This filtering procedure revealed that, of the 401 genes initially classified as flexible, 252 were actually part of the core, leaving 155 *bona fide* flexible genes. Before filtering, we observed gene content variation among control isolates, but these false positives were removed using the 1X coverage filter.

Inference of flexible gene origins

In order to estimate the origin of the flexible genome gene pool, we performed an extended phylogenetic analysis of all 155 flexible gene families. The flexible gene catalogue served as query for a blastp search against the NCBI database. For each gene, we selected the first 200 hits matching with an E-value below 1E-05. The hit sequences were aligned using muscle with default parameters [41] and gene trees were constructed with FastTree using default parameters [42]. We screened each gene tree to identify the closest relative sequences of each gene in our dataset. This allowed us to classify the flexible genes into three mutually exclusive categories: first, genes whose closest relative originated from the *Vibrio cholerae* gene pool; second, genes whose closest relative is from *Vibrio* but not *cholerae* (i.e non-cholera *Vibrio* species); and finally, the third category includes genes whose closest relative was outside the genus *Vibrio*. Trees were also displayed automatically using the FigTree java program for a manual inspection (http://tree.bio.ed.ac.uk/software/figtree/). To guard against false-positive inference of horizontal gene transfers from non-*V. cholerae*, a negative control was performed. We repeated the blastp/FastTree procedure using 155 genes extracted

randomly from core genes in our study. As expected, these were all assigned *Vibrio cholerae* taxonomic affiliations.

Molecular clock and demographic model comparison

For this analysis, we tested and compared both strict and uncorrelated lognormal molecular clock models and three coalescent models (exponential growth coalescent, constant-size coalescent and Bayesian Skyline demographic models), resulting in 6 possible model combinations. For all of them we used a GTR + G nucleotide substitution model and the sampling times for calibration. All of these combinations were run using 10,000,000 MCMC chains, with 10% burn-in and sampling every 5,000 generations. We used Tracer v.1.6 to ensure proper mixing, with all parameters having an effective sample size > 200. To select the best molecular clock and coalescent models, we estimate the marginal likelihoods for each combination via path-sampling, and we compared them with Bayes factors [9]. Divergence time, substitution rates and resulting tree were reported from the models with the highest marginal likelihood.

Tests for natural selection between patients

To distinguish between positive selection, purifying selection, or neutral evolution of protein-coding sequences, we considered variation in the proportion of non-synonymous hqSNVs (p_{NS}) in the *V. cholerae* core genome. Specifically, we evaluated how p_{NS} varied over time (a three-year period from 2011 to 2013) and among branches of the phylogenetic tree. We considered N=136 hqSNVs (excluding the ICE region, a mutation hotspot; Fig. S1) that varied among 21 isolates sampled over three years in Bangladesh (patients TC01-TC21) and the 122 isolates sampled from five patients from

Bangladesh (patients B1-B5) and three from Haiti (Patient H1-H3). For these analyses, we excluded iSNVs by considering only the most frequent haplotype found within each patient, assumed to be ancestral.

We first tested whether the overall observed p_{NS} is to be expected under a simple neutral model of evolution. We performed 1,000 simulations of N mutations randomly distributed across the core genome of the MJ1236 reference. For each simulation, we re-estimated the relative proportions of intergenic (p_I), synonymous (p_S) and non-synonymous (p_{NS}) mutations, using annotations available for MJ1236 (GenBank: CP001485-6). We controlled for potential effects of genome-wide nucleotide composition by comparing simulations with or without imposed GC content of mutated positions (i.e. matching the GC content observed among the N real hqSNVs). We also controlled for any bias in the transition:transversion ratio by comparing simulations with or without imposed transition rate (i.e. matching the ratio observed among the N real hqSNVs). We considered that the core genome evolved under positive selection when the observed p_{NS} was higher than in at least 97.5% of simulations. We considered that the core genome evolved under purifying selection when the observed p_{NS} was lower than in at least 97.5% of simulations. We failed to reject neutral evolution when the observed p_{NS} fell within the 95% range of simulations.

Second, we tested whether fixed core genome hqSNVs were distributed evenly across branches of the phylogeny. Specifically, we asked whether substitution rates differ between Bangladesh and Haiti, or between long internal branches and the shorter, more recent tips of the tree where selection may have had insufficient time to act. To do so, we defined 3 well-separated monophyletic clades based on the evolutionary tree of the *V. cholerae* core genome. The tree was built in MEGA5 using a maximum composite

likelihood model [10] (Fig. S7b). We distinguished hqSNVs that were fixed among clades (corresponding to long branches) from those that are variable within clades (the tips of the tree). We hypothesized that if differences in p_{NS} are observed between vs. within clades, this could suggest that selection or substitution rates vary among clades and over time. To test this, we performed 10,000 random permutations of hqSNVs among branches of the evolutionary tree, and for each permutation, we re-estimated p_I, p_S and p_{NS} within clade. For each monophyletic clade, we considered that the substitution rate was higher than expected by chance when the observed p_{NS} was higher than in at least 97.5% of permutations. We considered that the substitution rate was lower than expected by chance when the observed p_{NS} was lower than in at least 97.5% of permutations. We failed to reject neutral evolution when the observed p_{NS} fell within the 95% range of permutations.

Tests for natural selection within patients

To investigate the role of natural selection within versus between patients from Bangladesh and Haiti, we performed the McDonald-Kreitman test [11] to test the neutral hypothesis that nonsynonymous (NS) to synonymous (S) substitution ratios remained constant over evolutionary time (within vs. between hosts). Specifically, we computed the Fixation Index (equivalent to an odds ratio statistic) as the NS:S ratio between patients (fixed SNVs) divided by the NS:S ratio within patients (iSNVs). Significant deviations of the Fixation Index from neutral expectation were evaluated using Fisher's exact test.

We then tested whether iSNVs are equally distributed among patients, and if any patient contained an excess (possibly due to positive or balancing selection) or a deficit

(possibly due to efficient purifying selection) of NS iSNVs. To do so, we performed permutations of iSNVs among the eight patients and estimated expected iSNV frequencies (*F*) and p_{NS} per patient (B1-B5; H1-H3) and region (Bangladesh and Haiti). We first assigned each of the 122 isolates collected from the eight patients to one of the four following haplotypes: H_0 as isolates having the most frequent haplotype found within each patient, and assumed to be ancestral for that patient; H_{INT} as isolates having one intergenic iSNV; H_S as isolates having one synonymous iSNV and H_{NS} as isolates having one synonymous iSNV. (No isolates were observed with more than one iSNV, so these haplotypes are sufficient to model the observed intra-patient diversity). We then performed 10,000 random permutations of the four haplotypes among the 122 isolates. When an iSNV was shared between two or more isolates within a patient, but not observed in other patients, we ensured that these isolates were always assigned to the same patient during permutations. For each simulation and for each patient or region, we reported the total iSNV relative frequency (*F* = number of isolates containing iSNVs / the total number of isolates sequenced for that patient or region) and p_{NS}, defined as above. For each region and each patient, we considered that *F* and p_{NS} were higher or lower than expected by chance when the observed values were respectively higher or lower than in at least 97.5% of permutations. We concluded that *F* and p_{NS} were consistent with our neutral model when they fell within the 95% range of permutations.

Sensor histidine kinase protein conservation analysis

Of the three NS iSNVs detected in patient H1, two occur in the same gene, a predicted sensory histidine kinase (NCBI accession number ACQ61177, from the reference genome MJ-1236). We sought to determine whether these two NS iSNVs

occurred in conserved or variable peptides. To do so, we retrieved the 500 best matches (top BLAST hits) for the ACQ61177 protein sequence in NCBI GenBank using BLASTp. From these 500 homologous sequences, we removed identical (duplicate) sequences, and those that were truncated at the N or the C terminus, resulting in 465 unique homologous sequences. We then determined whether 4-amino-acid (4aa) peptides surrounding the mutated residues were conserved among these sequences. We defined a simple conservation score as the proportion of homologs having the reference peptide (from *V. cholerae* MJ1236). This score could be influenced by a biased sample of sequences in GenBank, and thus represents a rough estimate of conservation. In order to minimize the effect of peptide convergence, we did not consider 4aa motifs that were found at least twice in at least one sequence, which was not the case for any of the peptide motifs affected by the two observed iSNVs.

Supplementary Note

Natural selection is not detectable in *V. cholerae* core genome over a three-year period.

The constant molecular clock of the *V. cholerae* core genome (Table S3) suggests that the substitution rate was constant over our three-year survey. This constant substitution rate may hide more complex evolutionary processes, like natural selection on protein-coding sequences occurring before and during host infection, which could result in an overall excess (positive selection) or a deficit (purifying selection) of non-synonymous (NS) mutations. We considered only SNVs fixed among hosts. Among the 136 hqSNVs identified in the core genome (iSNVs excluded), 88 were identified as NS mutations (percentage NS, p_{NS} = 64.7%), based on the annotation of the MJ1236 reference genome. We then asked whether these mutations represent a random subset of expected mutations in the MJ1236 reference genome under neutral evolution. To test this, we simulated 1,000 sets of 136 mutations distributed randomly along the MJ1236 core genome and, for each of these sets, calculated the percentage of NS mutations. We took the mean p_{NS} across these simulations as our estimate of the expected p_{NS} under neutral evolution (see Material and Methods). We observed no significant difference between the observed (64.7%) and the expected p_{NS} (66.4±4.1%, $p>0.05$; Fig. S7b). We observed significantly higher GC content at mutated positions (72.8%) and higher transition rate (75%) than expected by chance (47.2% and 33.3%, respectively; $p<0.001$; 1,000 simulations). However, the difference between the observed and expected p_{NS} remained non-significant when incorporating the observed bias in GC content into the simulation (expected p_{NS} = 66.8±3.9%, $p>0.05$). Similarly, the

observed p_{NS} was not significantly different than expected when the observed bias in transition rate (expected p_{NS} = 60.3±4.2%, p>0.05), or both GC and transition biases together were incorporated into the model (expected p_{NS} = 60.9±4.2%, p>0.05; Fig. S7a). According to these results, we could not reject the hypothesis that protein-coding sequences in the *V. cholerae* core genome evolved under a neutral regime over a three-year period.

We next asked whether fixed core genome SNVs were distributed evenly across branches of the phylogeny. Specifically, we asked if substitution rates differ between Bangladesh and Haiti, or between long internal branches and the shorter, more recent tips of the tree where selection may have had insufficient time to act. To address these questions, we first defined three well-separated monophyletic clades based on the evolutionary tree of the *V. cholerae* core genome (Fig. 3), and corresponding to isolates from Haiti, isolates from Bangladesh sampled before October 2011 (BGD-B) and isolates from Bangladesh sampled after October 2011 (BGD-A). We distinguished hqSNVs that were fixed among clades (corresponding to long branches) from those that are variable within clades, the latter category corresponding to more recent mutations event (Fig. 3). If differences in p_{NS} are observed among these clades, this could suggest that selection or substitution rates vary among clades and over time. To test this, we permuted SNVs among branches of the evolutionary tree and recalculated p_{NS} within and between clades. We observed no significant difference in p_{NS} among clades (p > 0.05; 10,000 permutations; Fig. S7b), indicating that there is no evidence for differential selection on protein sequences among clades or over time.

Supplementary Figures

Fig. S1. hqSNV distribution across the *V. cholerae* genome. The x-axis indicates the position in the MJ1236 reference genome (reverse complement). The height of grey dots on the y-axis indicate the number of hqSNVs per 1kb discrete window, which is higher in the Integrative Conjugative Element (ICE; black region). Coloured circles indicate iSNVs identified in Bangladeshi (red) and Haitian (green) isolates. Circle diameter is proportional to iSNV frequency per 2kb window (ranging from 1 to 2 iSNVs per 2kb).

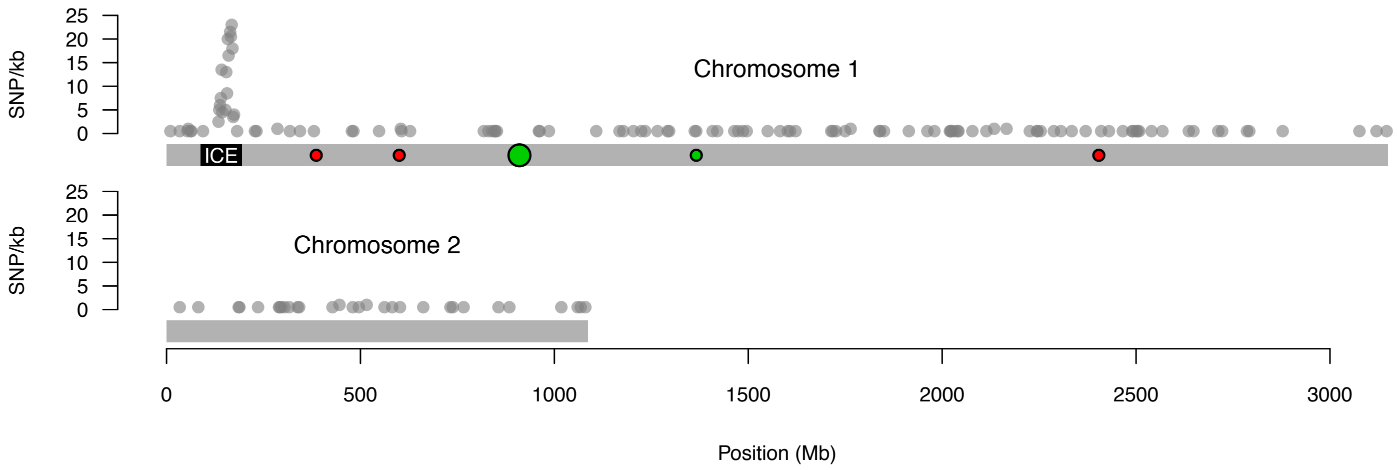

Fig. S2. Flexible genome analysis pipeline. (**a**) From sequence reads to primary flexible genome analysis. (**b**) From a flexible gene catalogue to a final picture of the flexible genome.

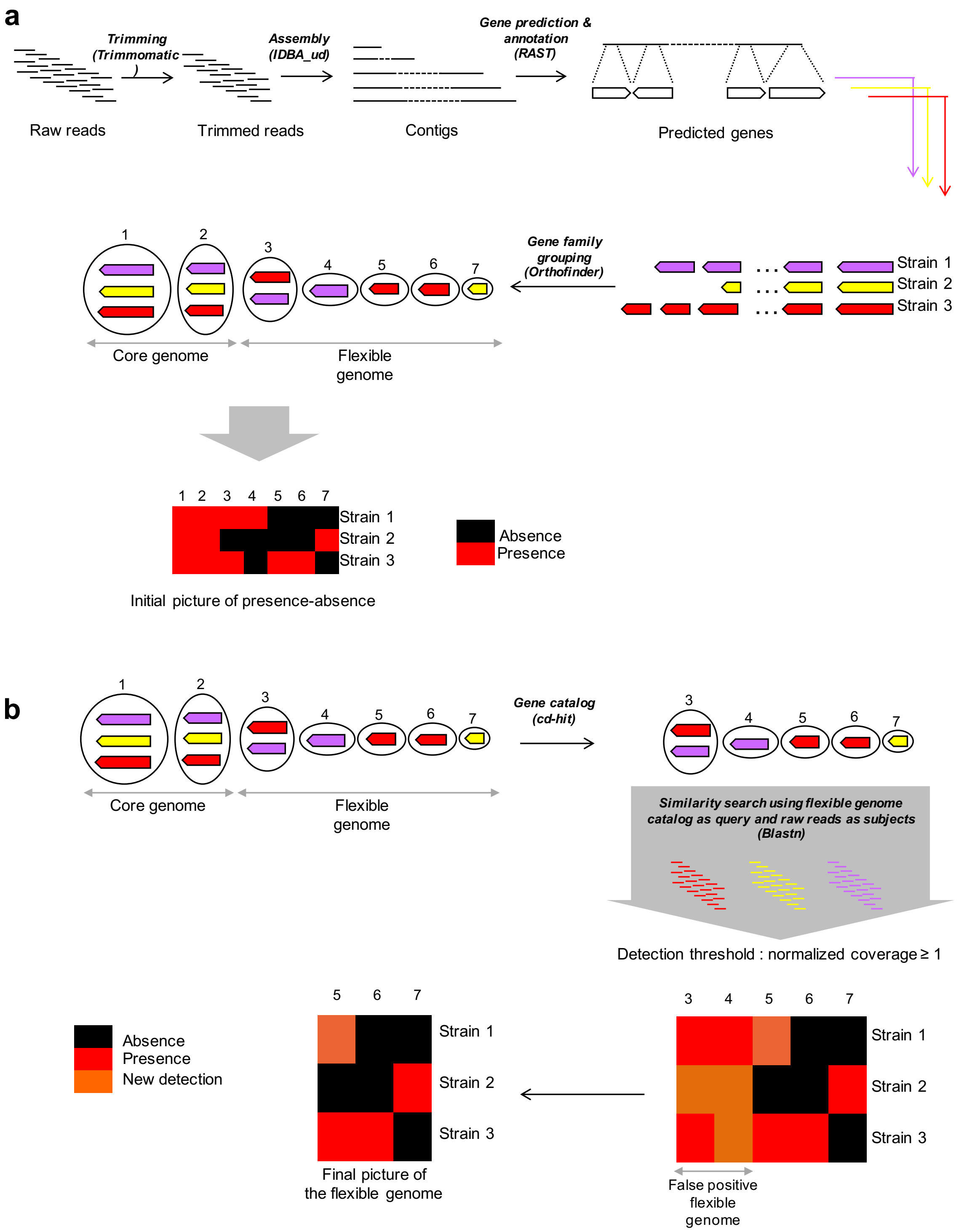

Fig. S3. Coverage filters used to infer presence-absence of 401 putative flexible genes. False positive flexible genes (green) are detected in all isolates (at a given coverage threshold), and are therefore part of the core, not the flexible genome. False negatives are genes known to be present in at least one genome (because they are part of the gene catalogue; Fig. S2) but are not identified in any genome at a given coverage threshold. Coverage is defined as the average coverage of a gene by sequence reads (Methods). Based on this analysis, we used a coverage filter of 1X to remove false positives, without suffering from false negatives.

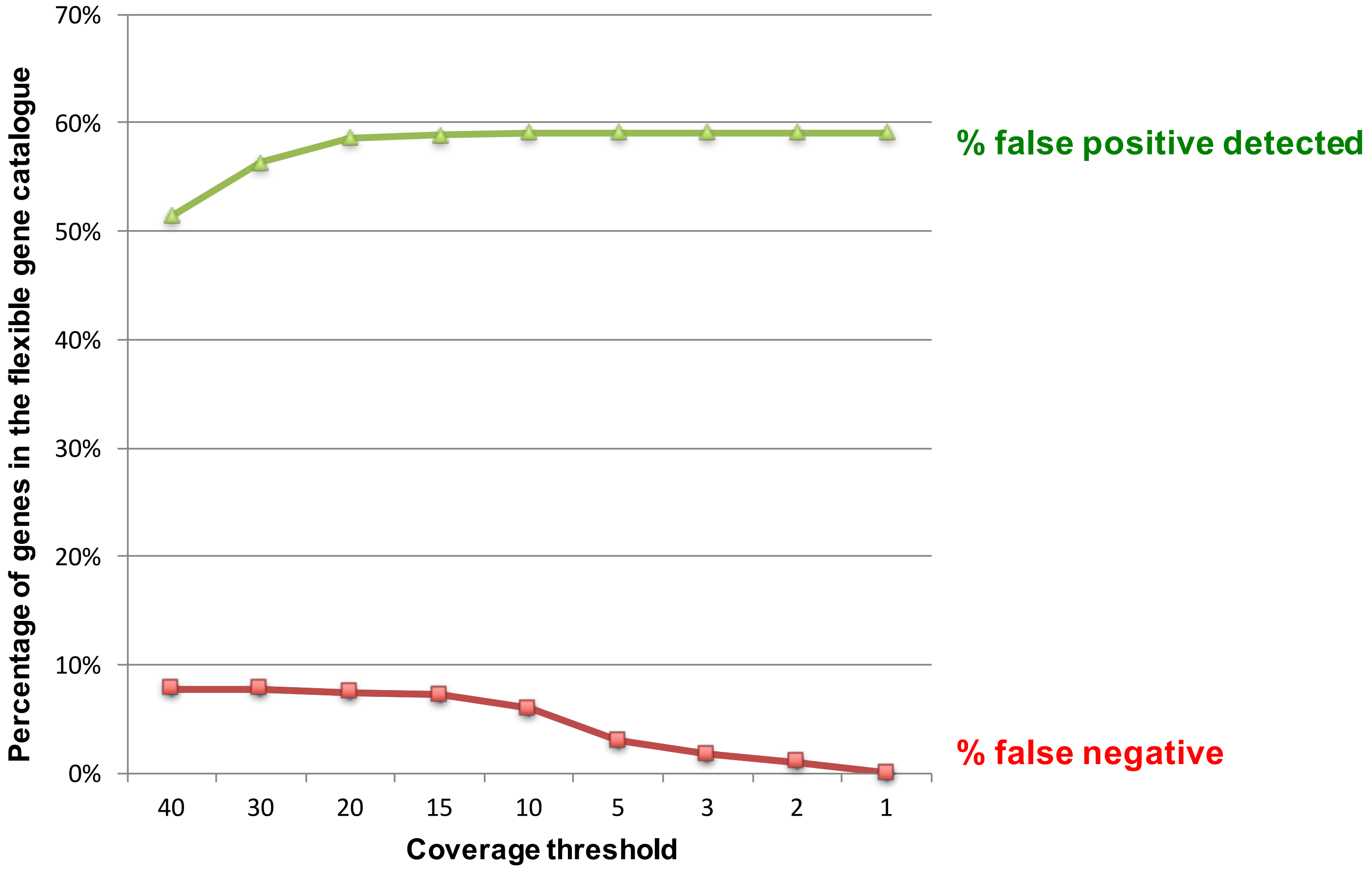

Fig. S4. Positive significant correlation between root-to-tip distance and year of sampling. Regression of the root-to-tip genetic distance as a function of sampling time (year) for 35 *V. cholerae* isolates sampled from 2011 to 2013. Each point corresponds to a genotype, the blue dashed line represents the linear regression and in grey its 95% confidence interval. *** indicates linear regression *P*-value < 0.0001.

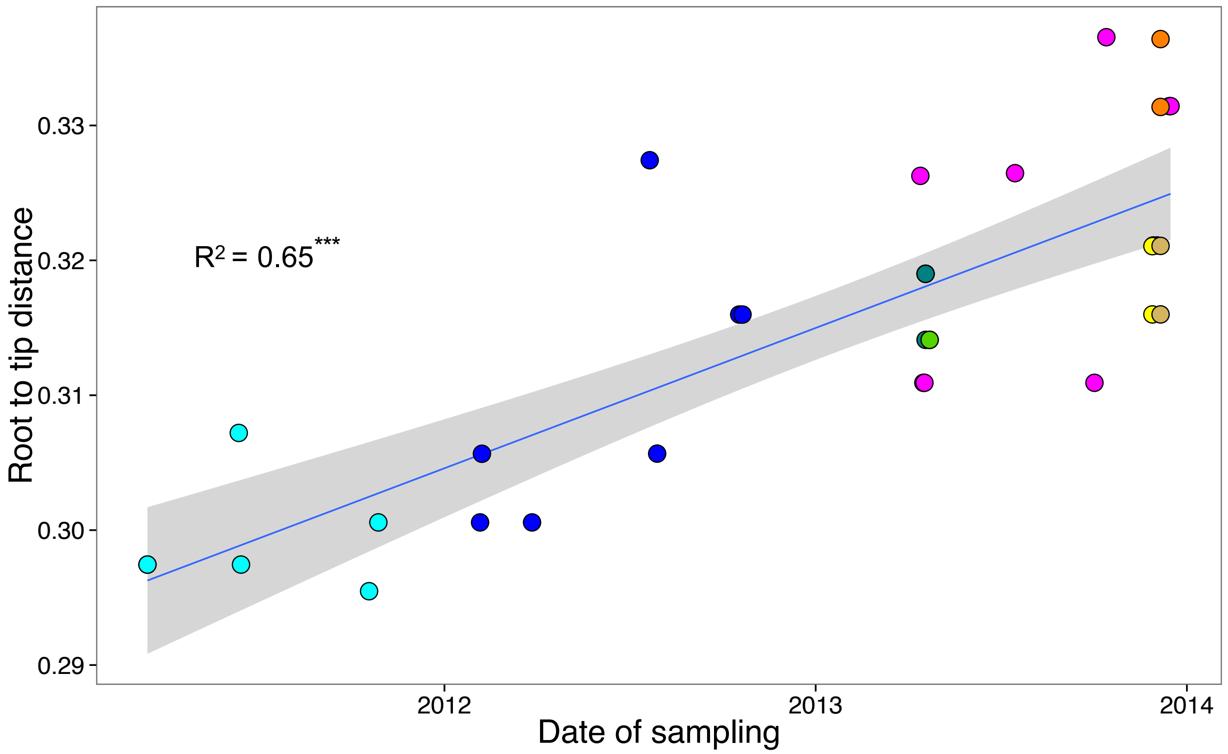

Fig. S5. Detailed presence/absence profile of gene families for each patient. The flexible genes within each patient are shown, with each patient in a separate panel. Only genes that vary in presence/absence among isolates (y-axis) from a given patient are shown (x-axis) Color denotes gene presence; black denotes absence. Duplicate isolates sequenced with two different procedures (Nextera/Miseq or NEB/HiSeq) are shown with a "b" (indicating the NEB/HiSeq procedure). All other isolates were sequenced with only one procedure (Table S5). Gene annotations are provided in Table S4.

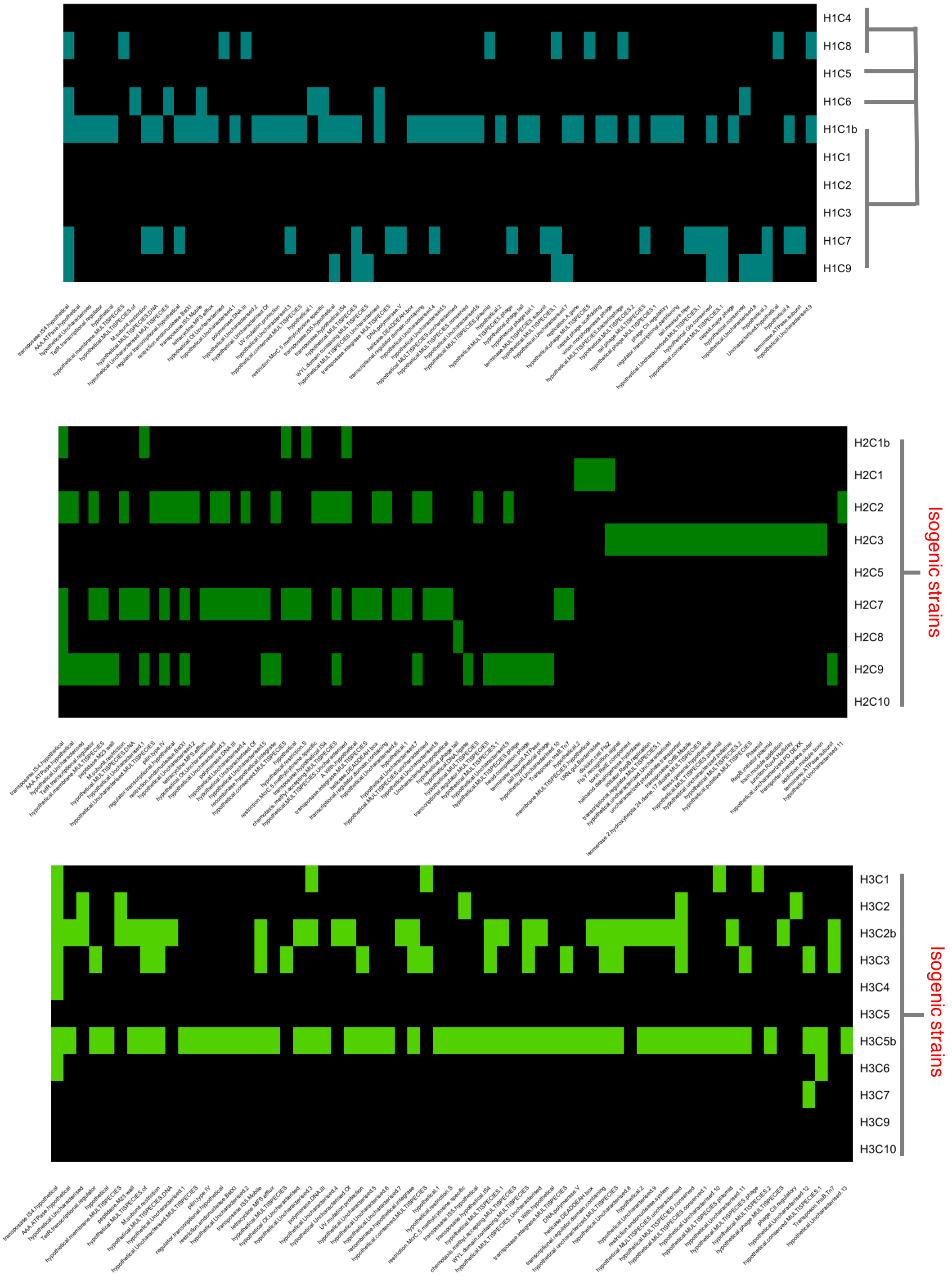

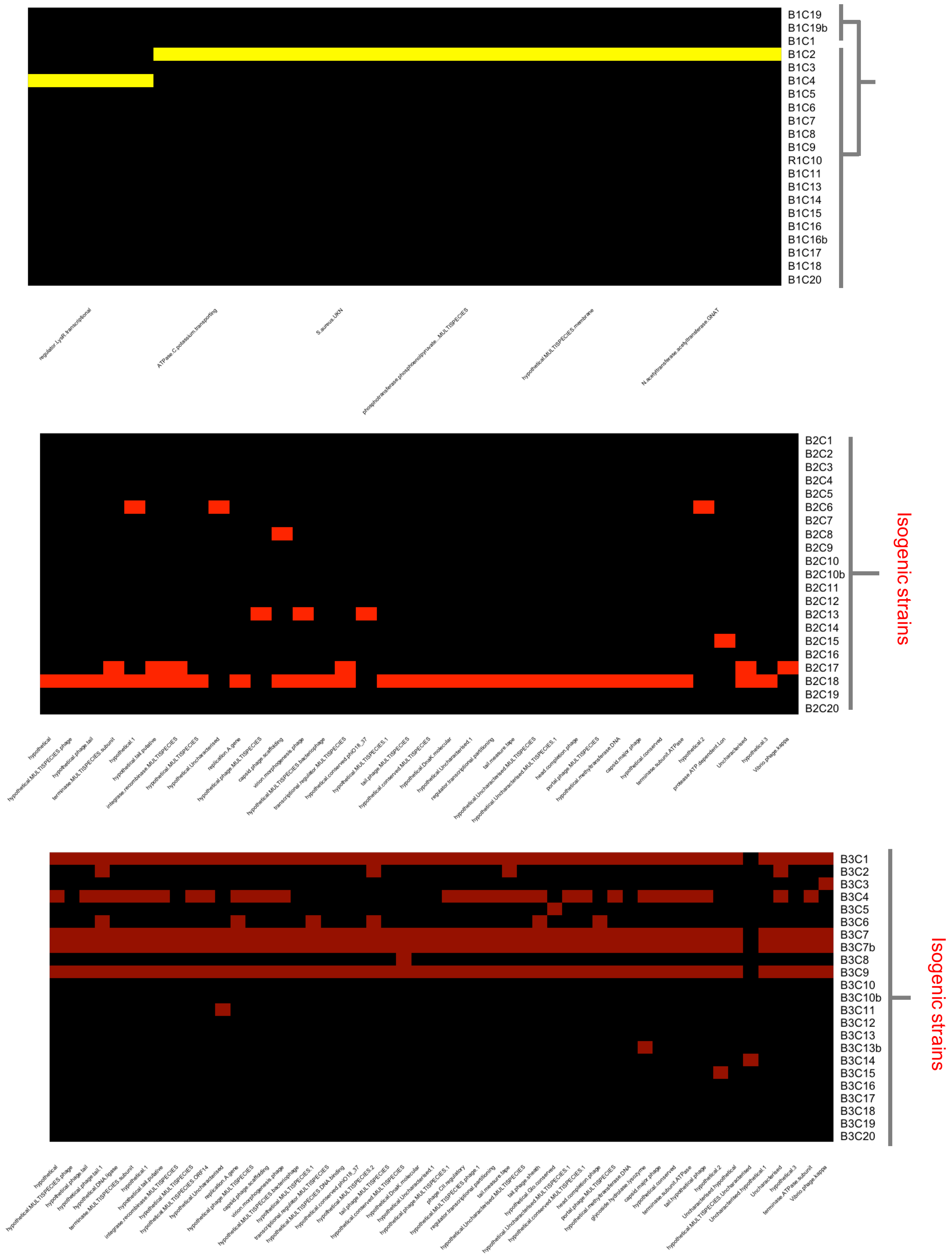
B1C19
B1C19b
B1C1
B1C2
B1C3
B1C4
B1C5
B1C6
B1C7
B1C8
B1C9
R1C10
B1C11
B1C13
B1C14
B1C15
B1C16
B1C16b
B1C17
B1C18
B1C20
B2C1
B2C2
B2C3
B2C4
B2C5
B2C6
B2C7
B2C8
B2C9
B2C10
B2C10b
B2C11
B2C12
B2C13
B2C14
B2C15
B2C16
B2C17
B2C18
B2C19
B2C20
Isogenic strains
B3C1
B3C2
B3C3
B3C4
B3C5
B3C6
B3C7
B3C7b
B3C8
B3C9
B3C10
B3C10b
B3C11
B3C12
B3C13
B3C13b
B3C14
B3C15
B3C16
B3C17
B3C18
B3C19
B3C20
Isogenic strains

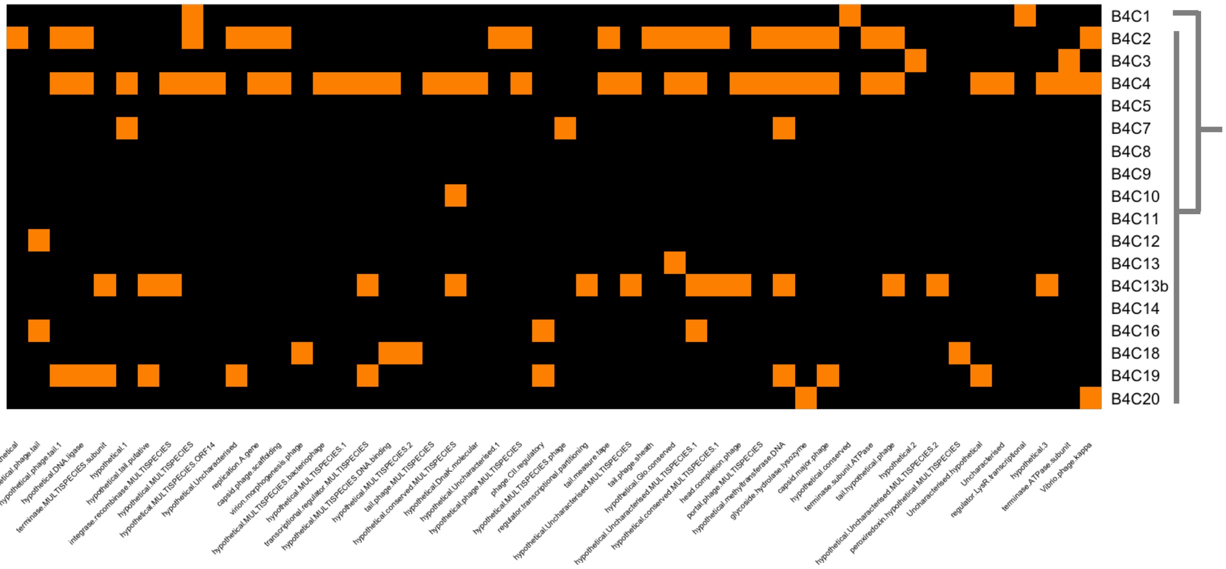

B4C1
B4C2
B4C3
B4C4
B4C5
B4C7
B4C8
B4C9
B4C10
B4C11
B4C12
B4C13
B4C13b
B4C14
B4C16
B4C18
B4C19
B4C20

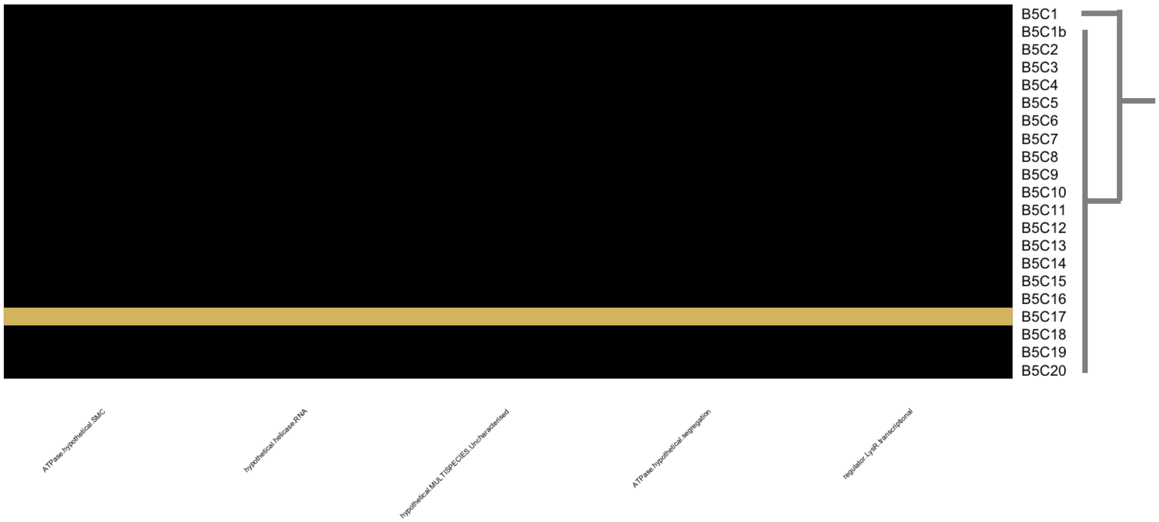

B5C1
B5C1b
B5C2
B5C3
B5C4
B5C5
B5C6
B5C7
B5C8
B5C9
B5C10
B5C11
B5C12
B5C13
B5C14
B5C15
B5C16
B5C17
B5C18
B5C19
B5C20
ATPase.hypothetical.SMC
hypothetical.helicase.RNA
hypothetical.MULTISPECIES.Uncharacterised
ATPase.hypothetical.segregation
regulator.LysR.transcriptional

Fig. S6. Non-synonymous iSNVs in a sensor histidine kinase in patient H1 affect highly conserved peptides. Distribution of peptide conservation scores (grey line; 4-amino-acid sliding windows) across the ACQ61177 protein sequence based on the comparison of 465 homologous proteins. The conservation score is defined as the proportion of homologs having the reference peptide (from *V. cholerae* MJ-1236). Grey dots indicate peptides that are not redundant across a single sequence. Red dots indicate peptides that are affected by NS iSNVs in patient H1. For each iSNV, details of the amino-acid reference (MJ1236) and mutated (H1C5 and H1C6) sequences are given for peptide with the highest conservation score (boxes).

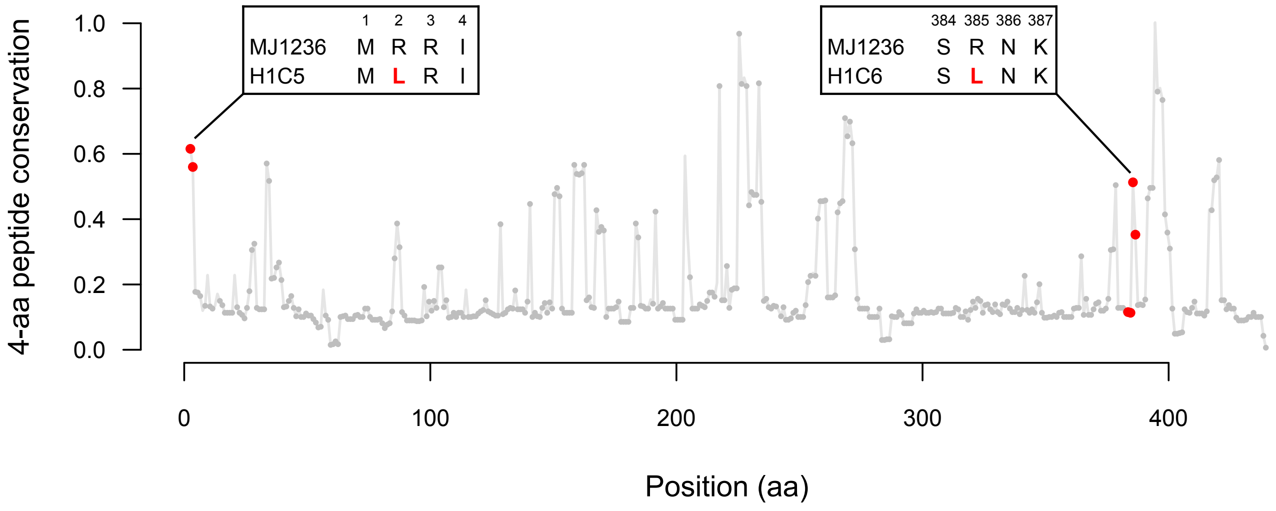

Fig. S7. Neutral evolution of protein-coding sequences in the *V. cholerae* core genome over a three-year period. These analyses are based on 136 hqSNVs identified among 29 isolates (iSNVs excluded, and excluding SNVs in the ICE). (A) The numbers of intergenic (I), synonymous (S) and non-synonymous (NS) mutations observed among the 136 hqSNVs (black bars) are no different than numbers expected after random mutation of the MJ1236 reference genome, simulated under four different models (see legend on the top and Methods for details of simulations). For each model, bars represent the expected average values and error bars show standard deviations calculated over 1,000 simulations. (B) Three well-separated clades (BGD-A, BGD-B and Haiti) are supported by an evolutionary tree based on 136 hqSNVs. The scale represents the number of single nucleotide substitutions per site. Nodes supported by bootstrap values ≥98% are indicated in grey (Maximum composite likelihood, bootstrap test, 1,000 replicates). (C) The observed numbers (black bars) of I, S, and NS mutations fixed (between clades) and variable (within clades) are not significantly different than expected by chance (grey bars). Grey bars and errors bars represent average and standard deviation of I, S and NS mutations expected from 10,000 random permutations of hqSNVs across the tree.

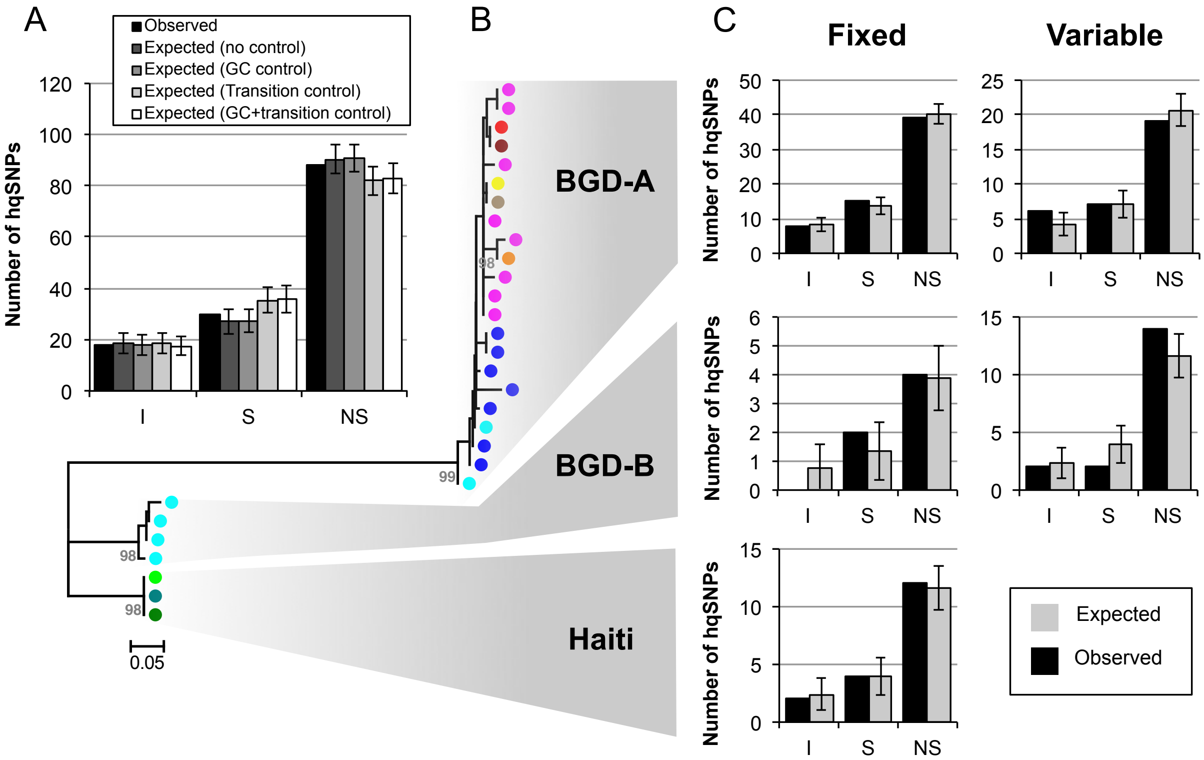
A
Observed
Expected (no control)
Expected (GC control)
Expected (Transition control)
Expected (GC+transition control)
Number of hqSNPs
0
20
40
60
80
100
120
I
S
NS
B
BGD-A
BGD-B
Haiti
98
99
98
98
0.05
C
Fixed
Variable
Number of hqSNPs
I
S
NS
Expected
Observed

Supplementary tables

Table S2. Estimation of effective population size (N_e) within each patient based on iSNVs numbers and frequencies.

Patients	#iSNVs	Patient allele frequency	Ne(S)	Ne(π)
B2 – B3 – H1 – H2	0	0	~0	~0
B1	1	0	44.12	16.5
B4	1	1/19	45	20
B5	1	1/20	43.3	15
H1	2	1/9<br>2/9	110.3	100

Table S3. Model comparison using path sampling to compute marginal likelihood estimations (MLE).

	Path-sampling log MLE	Model 1	Model 2	Model 3	Model 4	Model 5	Model 6
Model 1	-5722274,221	-	10.81	-0.47	-1027.76	-286.81	-742.66
Model 2	-5722285,028	-10.81	-	-11.28	-1038.57	-297.62	-753.47
Model 3	-5722273,752	0.47	11.28	-	-1027.29	-286.34	-742.20
Model 4	-5721246,462	1027.76	1038.57	-1027.29	-	740.95	285.09
Model 5	-5721987,408	286.81	297.62	-286.34	-740.95	-	-455.85
Model 6	-5721531,555	742.66	753.47	-742.20	-285.09	455.85	-

Higher MLE values indicate better model fit. Bayes factors are reported, with positive values indicating better relative model fit of the row's model compared with the column's model.

Model 1: Relaxed molecular clock, Bayesian skyline plot. Model 2: Relaxed molecular clock, Constant population size. Model 3: Relaxed molecular clock, Exponential population size. Model 4: Strict molecular clock, Bayesian skyline plot. Model 5: Strict molecular clock, Constant population size. Model 6: Strict molecular clock, Exponential population size.

Table S4. Flexible gene content variation within and between patients, according to sequencing methods.

Patients	#genes fixed within patients		#genes variable within patients	
	Nextera/MiSeq	NEBNext/HiSeq	Nextera/MiSeq	NEBNext/HiSeq
B1	111	111	0	6
B2	61	61	4	33
B3	61	61	50	51
B4	61	61	38	30
B5	111	111	0	5
H1	14	15	0	67
H2	14	14	62	31
H3	14	14	30	58

Table S6. Estimated divergence dates of *V. cholerae* sub-lineages and clusters.

Phylogenetic sub-lineages and clusters	Estimated divergence dates (day-month-year)	95% HPD interval	
BGD-A	17-05-2011	26-11-2010	05-10-2011
BGD-B	30-07-2010	09-12-2009	28-01-2011
Haiti - BDG-B	17-04-2006	24-04-2003	16-11-2008
Haiti	23-12-2012	03-09-2012	30-03-2013
Patient B1	22-10-2013	28-08-2013	26-11-2013
Patient B4	16-10-2013	11-08-2013	03-12-2013
Patient B5	27-10-2013	02-09-2013	04-01-2013
Patient H1	30-01-2013	05-11-2012	08-04-2013
Patient B2 - B3	07-11-2013	25-10-2013	28-11-2013

References

1. **Qadri F, Azim T, Chowdhury A, Hossain J, Sack RB, Albert MJ.** Production, characterization, and application of monoclonal antibodies to *Vibrio cholerae* O139 synonym Bengal. *Clin Diagn Lab Immunol* 1994;1: 51–54.

2. **Bolger AM, Lohse M, Usadel B.** Trimmomatic: a flexible trimmer for Illumina sequence data. *Bioinformatics* 2014;30: 2114–2120.

3. **Peng Y, Leung HCM, Yiu SM, Chin FYL.** IDBA-UD: a de novo assembler for single-cell and metagenomic sequencing data with highly uneven depth. *Bioinformatics* 2012;28: 1420–1428.

4. **Langmead B, Salzberg SL.** Fast gapped-read alignment with Bowtie 2. *Nat Methods* 2012;9: 357–359.

5. **Li H, Handsaker B, Wysoker A, Fennell T, Ruan J, Homer N, et al.** The Sequence Alignment/Map format and SAMtools. *Bioinformatics* 2009;25: 2078–2079.

6. **Rissman AI, Mau B, Biehl BS, Darling AE, Glasner JD, Perna NT.** Reordering contigs of draft genomes using the Mauve aligner. *Bioinformatics* 2009;25: 2071–2073.

7. **Garrison E, Marth G.** Haplotype-based variant detection from short-read sequencing. *arXiv preprint* arXiv:1207.3907*.* 2012

8. **Fu L, Niu B, Zhu Z, Wu S, Li W.** CD-HIT: accelerated for clustering the next-generation sequencing data. *Bioinformatics* 2012;28: 3150–3152.

9. **Baele G, Lemey P, Bedford T, Rambaut A, Suchard MA, Alekseyenko AV.** Improving the accuracy of demographic and molecular clock model comparison while accommodating phylogenetic uncertainty. *Mol Biol Evol* 2012;29: 2157–2167.

10. **Tamura K, Peterson D, Peterson N, Stecher G, Nei M, Kumar S.** MEGA5: Molecular Evolutionary Genetics Analysis Using Maximum Likelihood, Evolutionary Distance, and Maximum Parsimony Methods. *Mol Biol Evol* 2011;28: 2731–2739.

11. **McDonald JH, Kreitman M.** Adaptive protein evolution at the Adh locus in Drosophila. *Nature* 1991;20;351.6328:652.

12. **Aertsen A, Michiels CW.** Mrr instigates the SOS response after high pressure stress in *Escherichia coli*. *Mol Microbiol* 2005;58: 1381–1391.

13. **Aguilar-Rodríguez J, Sabater-Muñoz B, Montagud-Martínez R, Berlanga V, Alvarez-Ponce D, Wagner A, et al.** The Molecular Chaperone DnaK Is a Source of Mutational Robustness. *Genome Biol Evol* 2016;8: 2979–2991.

14. **Al-Maleki AR, Mariappan V, Vellasamy KM, Tay ST, Vadivelu J. Altered Proteome of Burkholderia pseudomallei** Colony Variants Induced by Exposure to Human Lung Epithelial Cells. *PLoS One* 2015;10: e0127398.

15. **Boin MA, Austin MJ, Häse CC.** Chemotaxis in *Vibrio cholerae*. *FEMS Microbiol Lett*

2004;239: 1–8.

16. **Breidenstein EBM, Janot L, Strehmel J, Fernandez L, Taylor PK, Kukavica-Ibrulj I, et al.** The Lon Protease Is Essential for Full Virulence in *Pseudomonas aeruginosa*. *PLoS* One 2012;7: e49123

17. **Cano DA, Pucciarelli MG, Portillo FG-D, Casadesús J.** Role of the RecBCD Recombination Pathway in *Salmonella Virulence*. *J Bacteriol* 2002;184: 592–595.

18. **Chiok KL, Addwebi T, Guard J, Shah DH**. Dimethyl Adenosine Transferase (KsgA) Deficiency in *Salmonella enterica* Serovar Enteritidis Confers Susceptibility to High Osmolarity and Virulence Attenuation in Chickens. *Appl Environ Microbiol* 2013;79: 7857–7866.

19. **Dörr T, Davis BM, Waldor MK.** Endopeptidase-mediated beta lactam tolerance. *PLoS pathog* 2015;17;11(4):e1004850.

20. **Favrot L, Blanchard JS, Vergnolle O.** Bacterial GCN5-related N-acetyltransferases: From resistance to regulation. *Biochemistry* 2016;55(7):989-1002.

21. **Heung LJ, Del Poeta M.** Unlocking the DEAD-box: a key to cryptococcal virulence? *J Clin Invest* 2005;115: 593–595.

22. **Houben ENG, Korotkov KV, Bitter W.** Take five — Type VII secretion systems of Mycobacteria. *Biochim Biophys Acta* 2014;1843: 1707–1716.

23. **Jurėnaitė M, Markuckas A, Sužiedėlienė E.** Identification and Characterization of Type II Toxin-Antitoxin Systems in the Opportunistic Pathogen *Acinetobacter baumannii*. *J Bacteriol* 2013;195: 3165–3172.

24. **Kaihami GH, de Almeida JRF, Santos dos SS, Netto LES, de Almeida SR, Baldini RL.** Involvement of a 1-Cys Peroxiredoxin in Bacterial Virulence. *PLoS Pathog* 2014;10: e1004442.

25. **Lin T, Gao L, Edmondson DG, Jacobs MB, Philipp MT, Norris SJ.** Central Role of the Holliday Junction Helicase RuvAB in vlsE Recombination and Infectivity of *Borrelia burgdorferi. PLoS Pathog* 2009;5: e1000679.

26. **Margolin W.** FtsZ and the division of prokaryotic cells and organelles. *Nat Rev Molecular Cell Biology*. 2005;6: 862–871.

27. **Martins-Pinheiro M, Marques RC, Menck CF.** Genome analysis of DNA repair genes in the alpha proteobacterium *Caulobacter crescentus*. *BMC microbiol* 2007;12;7(1):17

28. **Norton MD, Spilkia AJ, Godoy VG.** Antibiotic Resistance Acquired through a DNA Damage-Inducible Response in *Acinetobacter baumannii*. *J Bacteriol* 2013;195: 1335–1345.

29. **O'Boyle N, Houeix B, Kilcoyne M, Joshi L, Boyd A.** The MSHA pilus of *Vibrio parahaemolyticus* has lectin functionality and enables TTSS-mediated pathogenicity. *Int J Med Microbiol* 2013;303: 563–573.

30. **O'May GA, Jacobsen SM, Longwell M, Stoodley P, Mobley HLT, Shirtliff ME.** The high-affinity phosphate transporter Pst in *Proteus mirabilis* HI4320 and its importance in biofilm formation. *Microbiol* 2009;155: 1523–1535.

31. **Parsot C, Mekalanos JJ.** Expression of the *Vibrio cholerae* gene encoding aldehyde dehydrogenase is under control of ToxR, the cholera toxin transcriptional activator. *J Bacteriol* 1991;173: 2842–2851.

32. **Picardeau M.** Virulence of the zoonotic agent of leptospirosis: still terra incognita?. *Nat Rev Microbiol* 2017 ;1;15(5):297-307.

33. **Rektorschek M, Buhmann A, Weeks D, Schwan D, Bensch KW, Eskandari S, Scott D, Sachs G, Melchers K.** Acid resistance of *Helicobacter pylori* depends on the UreI membrane protein and an inner membrane proton barrier. *Molecular microbiology*. 2000;1;36(1):141-52.

34. **Rosch JW, Vega LA, Beyer JM, Lin A, Caparon MG.** The Signal Recognition Particle Pathway Is Required for Virulence in *Streptococcus pyogenes*. *Infect Immun* 2008;76: 2612–2619.

35. **Sala A, Bordes P, Genevaux P.** Multiple toxin-antitoxin systems in *Mycobacterium tuberculosis*. *Toxins* 2014;6;6(3):1002-20. doi:10.3390/toxins6031002

36. **Sheehan LM, Budnick JA, Blanchard C, Dunman PM, Caswell CC.** A LysR-family transcriptional regulator required for virulence in *Brucella abortus* is highly conserved among the α-proteobacteria. *Mol microbiol* 2015;1;98(2):318-28.

37. **Shin SJ, Wu C-W, Steinberg H, Talaat AM.** Identification of Novel Virulence Determinants in *Mycobacterium paratuberculosis* by Screening a Library of Insertional Mutants. *Infect Immun* 2006;74: 3825–3833.

38. **Szabady RL, Yanta JH, Halladin DK, Schofield MJ, Welch RA.** TagA is a secreted protease of *Vibrio cholerae* that specifically cleaves mucin glycoproteins. *Microbiol* 2011;157: 516–525.

39. **Talà A, Progida C, De Stefano M, Cogli L, Spinosa MR, Bucci C, et al.** The HrpB–HrpA two-partner secretion system is essential for intracellular survival of *Neisseria meningitidis*. *Cell Microbiol* 2008;10: 2461–2482.

40. **Tark M, Tover A, Tarassova K, Tegova R, Kivi G, Hõrak R, et al.** A DNA Polymerase V Homologue Encoded by TOL Plasmid pWW0 Confers Evolutionary Fitness on *Pseudomonas putida* under Conditions of Environmental Stress. *J Bacteriol* 2005;187: 5203–5213.

41. **Tribble GD, Mao S, James CE, Lamont RJ.** A *Porphyromonas gingivalis* haloacid dehalogenase family phosphatase interacts with human phosphoproteins and is important for invasion. *Proc Natl Acad Sci USA* 2006;103: 11027–11032.

42. **Vasu K, Nagaraja V.** Diverse functions of restriction-modification systems in addition to cellular defense. *Microbiol Mol Biol Rev* 2013;1;77(1):53-72.

43. **Venkatesh J, Kumar P, Krishna PSM, Manjunath R, Varshney U**. Importance of Uracil DNA Glycosylase in *Pseudomonas aeruginosa* and *Mycobacterium smegmatis*, G+C-rich Bacteria, in Mutation Prevention, Tolerance to Acidified Nitrite, and Endurance in Mouse Macrophages. *J Biol Chem* 2003;278: 24350–24358.

44. **Wang D, Guo C, Gu L, Zhang X.** Comparative study of the marR genes within the family Enterobacteriaceae. *J Microbiol* 2014;1;52(6):452.

45. **Wang Q, Millet YA, Chao MC, Sasabe J, Davis BM, Waldor MK.** A Genome-Wide Screen Reveals that the *Vibrio cholerae* Phosphoenolpyruvate Phosphotransferase System Modulates Virulence Gene Expression. *Infect Immun* 2015;83: 3381–3395.
